# *Zoophycos* macroevolution since 541 Ma

**DOI:** 10.1038/srep14954

**Published:** 2015-10-09

**Authors:** Li-Jun Zhang, Ruo-Ying Fan, Yi-Ming Gong

**Affiliations:** 1 Key Laboratory of Biogenic Traces & Sedimentary Minerals of Henan Province, Henan Polytechnic University, Jiaozuo, 454003, China; 2 State Key Laboratory of Biogeology and Environmental Geology, School of Earth Sciences, China University of Geosciences, Wuhan, 430074, China

## Abstract

*Zoophycos* is one of the most complex and enigmatic trace fossils recorded in marine strata from Cambrian to Quaternary worldwide, which is invaluable for the study of Phanerozoic development of organism–environment interactions. Here we address and demonstrate the macroevolution of Phanerozoic *Zoophycos* by assembling 448 points in constructing the Phanerozoic *Zoophycos* database based on 291 papers from 1821 to 2015 and 180 specimens from Cambrian to Palaeogene. The comprehensive dataset reveals, for the first time, five peaks and six depressions in Phanerozoic *Zoophycos* occurrence frequency. Secondly, the palaeogeographical distribution of *Zoophycos* is closely associated with the supercontinent Pangaea shifting, independent of the latitude. Our data also attest that the bathymetrical shift of *Zoophycos* from the littoral–neritic to bathyal environments is synchronized with the tiering shift from shallow to deep. By detailed comparison with body fossils, geochemical and palaeogeographical records, we conclude that the macroevolution of Phanerozoic *Zoophycos* is multi-affected by the global biodiversity expansion, benthic nutrient enhancement, and the biotic macroevolution of the *Zoophycos*-producers. The macroevolution of development evidenced by the morphological changes of *Zoophycos* and other trace fossils, may have great implications on the behavioural and physiological adaptation of ancient animals to the environments.

*Zoophycos* is one of the most complex, enigmatic and widespread trace fossils in Phanerozoic marine environments^1^,^2^,^3^,^4^,^5^. *Zoophycos* was initially named as a plant genus by Massalongo^6^,^7^. Since then, several morphological types, like J- or U-forms^2^, and ethologies, such as surface detritus-feeding^8^, refuse dump^4^,^9^, cache^4^,^10^, deposit-feeding^2^,^11^,^12^, and gardening^5^,^9^, have been proposed for it. There is still no consensus concerning the taxonomy of *Zoophycos* and related forms. Most researchers favour the term ‘*Zoophycos* Group’^13^ to embrace these complex structures.

*Zoophycos* has been speculated to evolve from simple to complex in morphology and from shallow to deep marine in environmental distribution during the Phanerozoic^1^,^3^,^14^,^15^. However, these studies largely dealt with *Zoophycos* reports only from Europe and America. Detailed studies of Phanerozoic major bio-environmental events in recent years^16^,^17^ ask for an integrated study of organism–environment interactions, combining body and trace fossils, geochemical, and palaeogeographical approaches. In this study, we established the Phanerozoic *Zoophycos* database based on a worldwide collection of literature and specimens (Table S1) and plotted the records together with Phanerozoic faunal and environmental changes. The comprehensive analysis opens up the opportunity to explore finer-scale macro-evolutionary pattern of Phanerozoic *Zoophycos*.

In this study, four major morphological constructs are used in describing *Zoophycos*: spreiten, primary lamellae, cylindrical tunnel/virtual central axis, and marginal tube, as well as three minor ones: secondary lamellae, number of whorls, and marginal lobes, as illustrated in well-preserved specimens (Fig. 1). The constructions of *Zoophycos* include both upwards and downwards^1^,^4^, but here we just show the downwards constructions in the three-dimensional modeling of *Zoophycos* by computer (Fig. 1).

## Results

### Morphological macroevolution of *Zoophycos*

**Figure 1:**
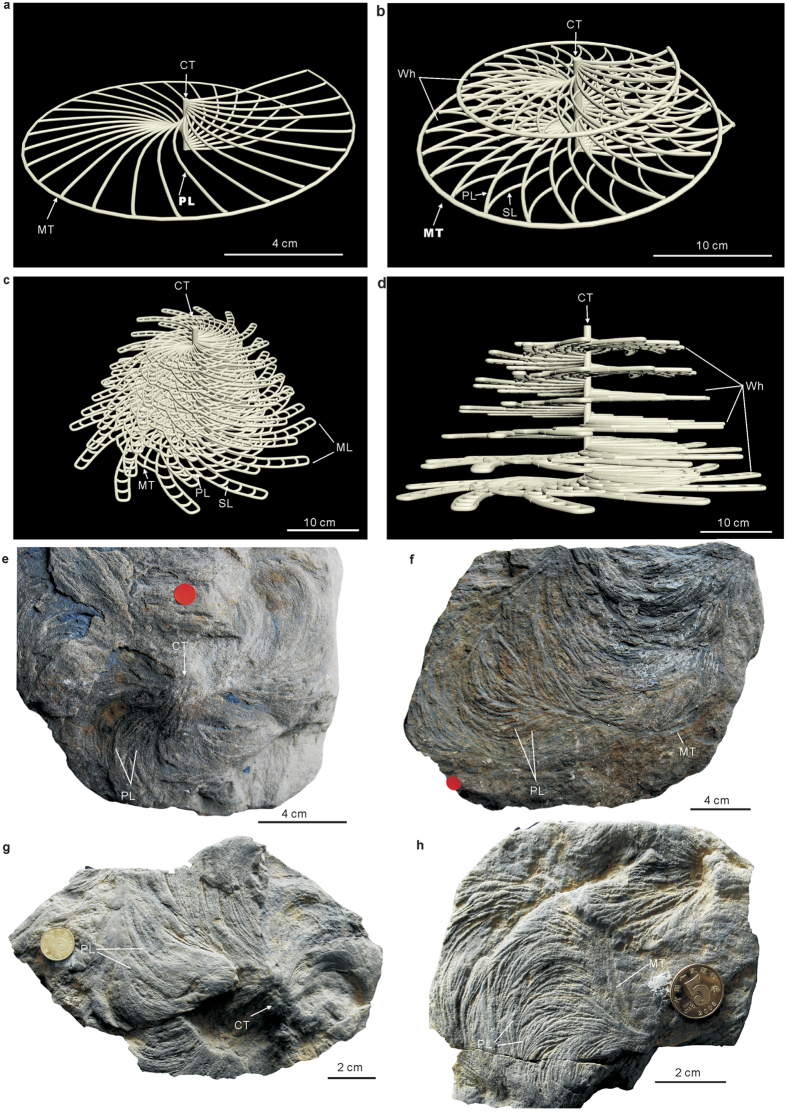
Morphology of *Zoophycos*. (**a**–**d**) Three-dimensional model of *Zoophycos*, including protrusive and retrusive spreiten, but here we just show the protrusive ones. (**a**) Palaeozoic *Zoophycos*. Simple, helicoidal circular spreiten with one whorl. (**b**) Mesozoic *Zoophycos*. Simple–complex, helicoidal circular spreiten with two–five whorls. (**c**,**d**) Cenozoic *Zoophycos*. Complex, helicoidal lobate spreiten with several whorls. (**e**–**h**) Examples of Devonian *Zoophycos*, helically coiled circular (**e**,**f**) or tougue-like (**g**,**h**) spreiten, with distinctive primary lamellae, marginal tube and cylindrical tunnel, secondary lamellae not well preserved. (**e**,**f**) *Zoophycos* from the Givetian Songjiaqiao Fm., Dushan, Guizhou, South China, upper bedding surface. (**g**,**h**) *Zoophycos* from the Givetian Tiaomajian Fm., Guanyang, Guangxi, South China, upper bedding surface. Abbreviation: CT = cylindrical tunnel, MT = marginal tube, PL = primary lamellae, SL = secondary lamellae, ML = marginal lobes, and Wh = whorls. (**a**–**d**) were designed and made by Li-Jun Zhang using the 3DMAX software, (**e**–**h**) were collected by Li-Jun Zhang and Yi-Ming Gong in the field and taken photos in the Paleontology Lab of State Key Laboratory of Biogeology and Environmental Geology by Li-Jun Zhang.

**Figure 2:**
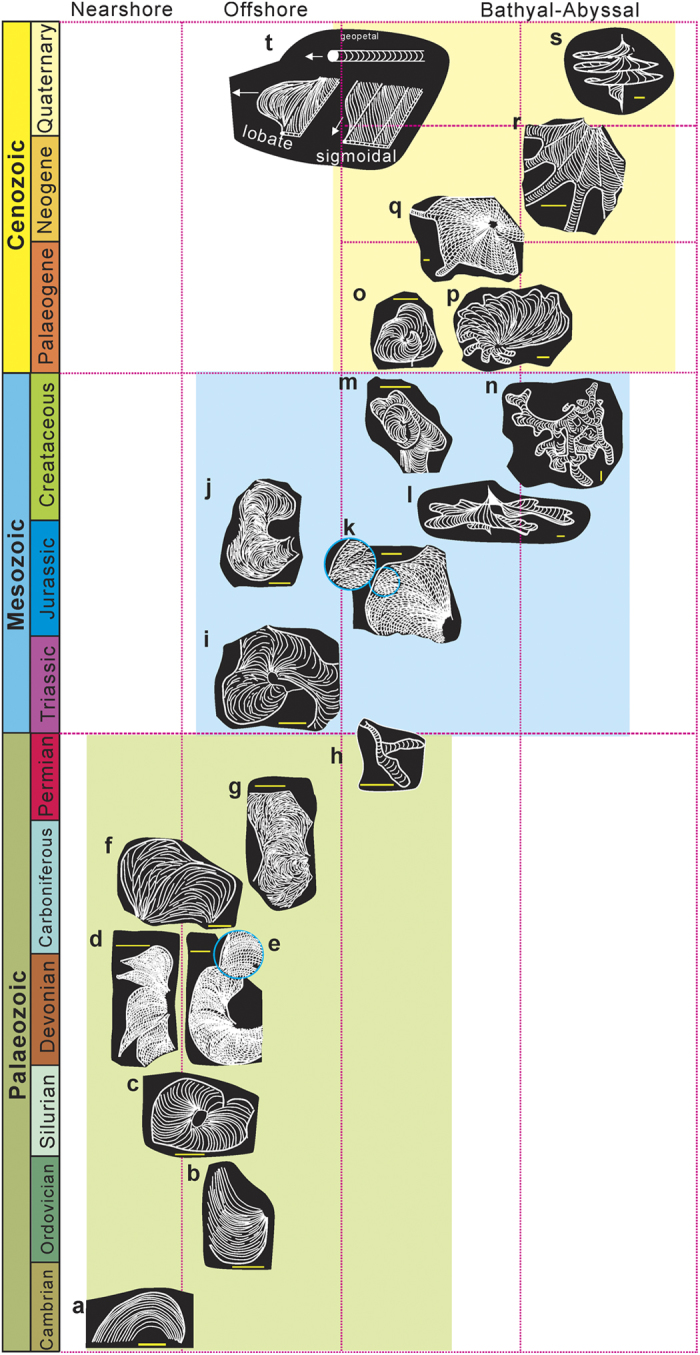
Morphological macroevolution of Phanerozoic *Zoophycos*. (**a**) cocks-tail shaped spreiten with J-shaped primary lamellae, Cambrian, USA^48^. (**b**) cocks-tail shaped spreiten with J-shaped primary lamellae, Ordovician, China^49^. (**c**) Spiraling circular spreiten of one whorl, Devonian, Bolivia^19^. (**d**) Centrifugal, alate spreiten, Devonian, Libya^14^. (**e**) Centrifugal, spiraling spreiten, Devonian, Libya^14^. (**f**) Spiraling tongue-like spreiten, Pennsylvanian, USA^50^. (**g**) Ambivalent backfilled spreiten, Pennsylvanian, Austria^14^. (**h**) Spiraling elongate lobate spreiten, Permian, China^23^. (**I**,**j**) Spreiten made of centripetal U-shaped protrusive burrows, Triassic and Jurassic, Germany^14^. (**k**) Irregular centripetal spiraling spreiten, Jurassic, France^14^. (**l**) Spiraling gently lobed circular spreiten, Jurassic, France^1^. (**m**) Spiraling lobate spreiten in slightly conical outline, Cretaceous^14^. (**n**) Irregular spiraling lobate spreiten, Cretaceous^51^. (**o**,**p**) Regular spiraling lobate spreiten, Eocene, Italy^14^,^51^. (**q**) *Rhizocorallium*-like spiraling spreiten, Oligocene, New Zealand^11^,^14^. (**r**) Irregular spiraling spreiten with long lobes, Miocene, Turkey^52^. (**s**) 3-D morphology of *Zoophycos* from Quaternary deep-sea deposits^10^. (**t**) trace making paradigm of *Zoophycos*, showing production of sigmoidal secondary lamellae and U-shaped protrusive burrows^14^. All scale bar, 5 cm.

Phanerozoic *Zoophycos* evolved from small, simple, helicoidal, rooster-shaped, circular/elliptical spreiten of one to two whorls without marginal lobes in the Palaeozoic, to large, complex, helicoidal, lobate spreiten of several whorls in the Cenozoic (Table 1 and Figs 1a–d and 2).

In the Palaeozoic, *Zoophycos* was composed of relatively simple, helicoidal, circular/elliptical spreiten, with pronounced primary lamellae, cylindrical tunnel and continuous and/or discontinuous marginal tube (such as *Zoophycos caudagalli*, *Zoophycos velum*). From the Cambrian to Devonian, *Zoophycos* were represented by simple circular/elliptical spreiten or tongue-like spreiten made of centrifugal (tending to move away from a center) U-shaped protrusive or J-shaped retrusive burrows with one whorl and evenly spaced primary lamellae (Fig. 2a–e). Carboniferous and Permian *Zoophycos* show irregular spreiten consisting of centrifugal U-shape protrusive or J-shaped retrusive burrows, exhibiting alternating light- and dark-coloured minor lamellae with different thickness in cross-section, in normally two whorls, and the secondary lamellae are commonly seen irregularly distributed between the primary lamellae on the bedding surface (Fig. 2f–h).

The shelf lineage of *Zoophycos* continued into the Triassic and Jurassic, displaying irregular spreiten made of centripetal (tending to move towards a center) U-shaped protrusive burrows with two to five whorls, secondary lamellae regularly distributed between primary lamellae (such as *Zoophycos* morphotype C^1^) (Fig. 2i–l). Cretaceous *Zoophycos* were commonly more than four whorls and characterized by irregular marginal lobes, with radiating primary lamellae and sigmoidal secondary lamellae^4^ (Fig. 2m,n), and continuous marginal tube.

In the Cenozoic, *Zoophycos* took the shape of complex, irregular skirt-like spreiten in several whorls with many long marginal lobes and alternating primary and secondary lamellae (such as *Zoophycos insignis*^18^, *Zoophycos rhodensis*^4^) (Fig. 2o–s), which are largely preserved in the upper part of sandy turbidites, silty turbidites or deep-sea oozes. The spreite is composed of alternate crescent light- and dark-coloured minor lamellae in cross-section, with or without faeces^8^.

The marginal tube is the vacant space (though may be later filled or compacted) created by the lateral shift of the *Zoophycos*-producers in constructing the spreiten. The width of the marginal tube hardly changed in the Phanerozoic, centring around 4 mm (Table 1). From current data, the width of the spreite gradually increases from 17.98 cm in the Lower Palaeozoic to 43.23 cm in the Cenozoic (Table 1).

### Tiering and bathymetrical shift of *Zoophycos*

**Figure 3:**
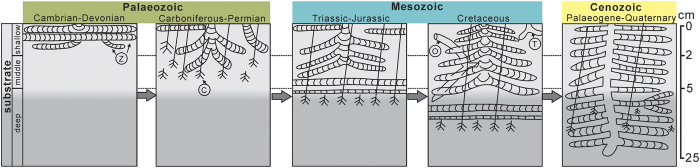
Tiering evolution of Phanerozoic *Zoophycos*. Z: *Zoophycos*; C: *Chondrites*; O: *Ophiomorpha*; T: *Thalassinoides*.

*Zoophycos* shifted gradually from the shallow to deep tiers of marine substrates in the Phanerozoic (Fig. 3). During the Cambrian–Devonian, *Zoophycos* predominantly occurred in the shallow-tier as thin planar spreite^19^. Since the Carboniferous, *Zoophycos* has been frequently found together with *Chondrites*—a typical middle–deep tier Chemichnion^20^. *Zoophycos* dominated the shallow–middle tier^21^ from the Carboniferous to Permian, and became completely restricted to the shallow–middle tier^1^,^15^ during the Triassic and Jurassic. Cretaceous *Zoophycos* was shallow–deep tiers^4^,^8^, coexisting with *Chondrites*, *Thalassinoides*, and *Ophiomorpha*. In the Palaeogene–Quaternary, *Zoophycos* mostly occurred in the deep tier with *Chondrites*^20^.

**Figure 4:**
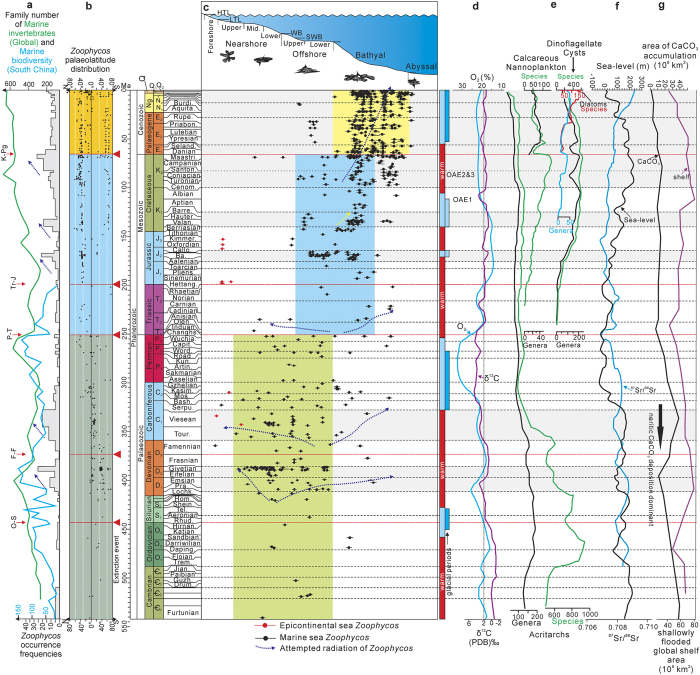
Spatiotemporal distribution of Phanerozoic *Zoophycos* and its bio-environmental background. (**a**) Curves of global family number of marine invertebrates^53^ and marine biodiversity from South China^54^. (**b**) Palaeolatitudinal distribution of *Zoophycos*. (**c**) Bathymetrical distribution of *Zoophycos*, the *Zoophycos* sketches redrawn from previous studies^1^,^10^,^24^, the green, blue, yellow column represent the main *Zoophycos* distribution areas in the Palaeozoic, Mesozoic and Cenozoic, respectively. (**d**) Column recording climate changes and glacial periods^55^, curves for atmospheric oxygen^38^ and δ^13^C^26^. (**e**) Acritarchs^37^, calcareous nanoplankton (genera^56^,^57^, species^58^), dinoflagellate cysts (genera^56^,^57^, species^59^) , and diatoms (genera^56^,^57^, species^57^) diversity curves^35^. (**f**) Curves of sea-level changes^45^,^60^ and ^87^Sr/^86^Sr ratios^26^. (**g**) Curves of areas of CaCO_3_ accumulation and shallowly flooded global shelf area^28^,^45^. Abbreviation: HTL = high-tide line, LTL = low-tide line, WB = wave base, and SWB = storm wave base. The grayish sections in the figure represent the five peaks of Phanerozoic *Zoophycos*.

Bathymetrically, the habitats of *Zoophycos* changed from the shelf in the Palaeozoic, lower shelf-slope in the Mesozoic, to the bathyal in the Cenozoic through three radiations (Fig. 4c). The first radiation started in the Ludlow–Pridoli and peaked in the neritic sea of the Middle Devonian. The second radiation took place after the Frasnian–Famennian boundary interval and maximized in the neritic sea of the Mississippian. The third radiation began after the end-Permian mass extinction, when *Zoophycos* started their Mesozoic migration and adaption to the bathyal environment.

### Occurrence frequencies and palaeolatitudinal distribution of *Zoophycos*

The occurrence frequencies of *Zoophycos* in the Phanerozoic demonstrate five peaks in the Middle Devonian, Mississippian, Middle Jurassic, Early Cretaceous, and Late Cretaceous, as well as six depressions during the Cambrian–Silurian, and at the Frasnian–Famennian, Carboniferous–Permian, Permian–Triassic, Triassic–Jurassic, and Jurassic–Cretaceous transitions (Fig. 4a). *Zoophycos* occurrence frequencies display rough correlation with the Global and South China marine invertebrate diversity changes and discrepancies exist on a finer scale (Fig. 4a). Notwithstanding the marine invertebrate radiation in the Early–Middle Ordovician, *Zoophycos* radiated and quickly reached peak only later in the Devonian^19^,^22^. In contrast with marine invertebrate radiation in the Carboniferous–Permian, there was only trivial radiation even recession of *Zoophycos* in the Permian. Nevertheless, the occurrence frequencies and radiations of Mesozoic and Cenozoic *Zoophycos* show well correlation with contemporaneous marine invertebrate diversity curves. Considering the big five mass extinctions, the *Zoophycos* occurrence frequencies were greatest impaired by the Frasnian–Famennian mass extinction and least or not at all affected by the other mass extinctions (Fig. 4a).

As for palaeolatitudinal distribution, Early Palaeozoic *Zoophycos* was concentrated in the Southern Hemisphere while Late Palaeozoic–Cenozoic *Zoophycos* occurred in both the Northern and Southern Hemispheres (Fig. 4b). Specifically, Cambrian–Mississippian *Zoophycos* was predominantly found in the low latitudes of the Southern Hemisphere^19^. In the Pennsylvanian and Permian, however, *Zoophycos* had already occupied the low latitudes of both the Northern and Southern Hemispheres^21^,^23^. A major transformation took place after the Permian–Triassic transition, when *Zoophycos* shifted considerably northward. Triassic–Jurassic *Zoophycos* was concentrated in the low–middle latitudes of the Northern Hemisphere^1^,^24^. Till the Middle Jurassic, *Zoophycos* was widely distributed in the middle latitude of the Northern Hemisphere^1^. *Zoophycos* expanded substantially in geographical distribution during the Cretaceous and Quaternary, occupying large areas of the middle latitude of the Northern Hemisphere and high latitude of the Southern Hemisphere^1^,^25^. These trends indicate that the palaeolatitudinal distribution of Phanerozoic *Zoophycos* was closely related to the Pangaea integration and disintegration.

## Discussion

### Role of mass extinctions and Pangaea

From the above evidences, the occurrence frequencies and palaeolatitudinal distribution of Phanerozoic *Zoophycos* not only demonstrate close links with the two macrofaunal radiations (Early–Middle Ordovician and Middle Triassic) and mass extinctions in the Phanerozoic but also show variance in the timing and scale of certain changes.

The first *Zoophycos* radiation was maximized in the neritic seas of the Middle Devonian. The main reasons why *Zoophycos* occurrence frequencies had not significantly elevated in the Middle Ordovician could be the oligotrophic marine environments^26^,^27^ and impending shrinkage of shallow marine niches^28^, as exemplified by the low ^87^Sr/^86^Sr ratios and reduced shelf area at that time (Fig. 4f,g). Marine substrates experienced fundamental changes in the Devonian, symbolized by the gradually increased ^87^Sr/^86^Sr and δ^13^C values (Fig. 4d,f). The development of deep-rooted plants in the Middle Devonian contributed to the establishment of effective biological weathering forming real soil^29^,^30^,^31^, introducing large quantities of land-based nutrients to oceans. Abundant acritarchs (Fig. 4e) also provided nutrients for the marine benthic communities in the Devonian. In addition, the increased shallow marine areas created abundant niches for the benthos (Fig. 4g). The radiation actually began in the late Silurian to either shallower or bathyal environments, and peaked in the Middle Devonian neritic seas (Fig. 4c). However, the Frasnian–Famennian mass extinction severely abated *Zoophycos*, marked by the first period of low occurrence frequencies of this ichnogenus.

The second radiation of *Zoophycos* occurred in the Famennian, also in two directions to either neritic^21^ or bathyal environments^32^, and ultimately peaked in the neritic seas of the Visean^22^. The revolution of marine fauna and substrates were accounted for the second radiation of *Zoophycos*. Through both the Frasnian–Famennian and Devonian–Carboniferous mass extinctions, new benthic fauna characterized by echinoderms and productoids^33^,^34^ appeared and became widespread. Meanwhile, the marine shelfal substrates also changed from clastics-dominated in the Devonian to carbonates-dominated in the Mississippian, evidenced by the rapidly increased areas of shelf CaCO_3_ accumulation, relatively high δ^13^C values and reduced ^87^Sr/^86^Sr ratios during the Mississippian (Fig. 4d,f,g).

The third *Zoophycos* radiation, either to neritic or bathyal environments, began in the Early to Middle Triassic, and finally peaked in the lower offshore and slope environments of the Middle Jurassic. The Permian drop of *Zoophycos* occurrence frequencies might have resulted from sea regression and reduced shelf areas due to the formation of the Pangaea (Fig. 4f,g). However, the Pangaea started to break up in the late Early Triassic, causing sea-level rise (Fig. 4f) and increase of shelf areas (Fig. 4g). On the other hand, the marine and terrestrial ecosystems revolutionized after the Permian–Triassic and Triassic–Jurassic mass extinctions, for instance the Palaeozoic marine fauna was replaced by the modern marine fauna composed predominantly of bivalves and gastropods^34^. The increased biodiversity and abundance of Mesozoic marine fauna accelerated the competition in the neritic seas, which might have forced the *Zoophycos*-producers and other benthic organisms such as brachiopods and crinoids to migrate to the bathyal environments. The third peak of *Zoophycos* occurrence frequencies was thus achieved in the slope environments of the Middle Jurassic.

The high occurrence frequencies of *Zoophycos* in the Cretaceous–Cenozoic were closely linked with the ever increased shelf areas (Fig. 4e,g), and with plankton blooms, most importantly coccolithes^35^, which enhanced the nutrient supply to the deep sea. Unlike the three aforementioned two-direction radiations, the radiations after the K–Pg mass extinction took only one direction from the shallow bathyal to deep bathyal. The scale of the three Palaeozoic and Early Mesozoic radiations were diminished in turn but the scale of Cretaceous–Cenozoic radiations increased stepwise (Fig. 4a,c). The *Zoophycos*-producers, after three radiations in the Palaeozoic and Early Mesozoic, had completed the migration and adaptation to the deep sea by the end of Mesozoic, and finally migrated and adapted to the bathyal environments in the Cenozoic.

In summary, the occurrence frequencies and palaeolatitudinal distribution of *Zoophycos* were mainly correlated with the Phanerozoic marine faunal changes and the shifting Pangaea. The mass extinctions and formation of Pangaea not only resulted in the decrease of marine invertebrate diversity, but also the low occurrence frequencies of *Zoophycos*. The contraction or expansion of shelf areas, successively increased competition in benthic marine invertebrates, and the Mesozoic–Cenozoic plankton blooms are all key factors in inducing the migration of *Zoophycos* to the bathyal substrates.

### Role of the increasing marine biodiversity

The bathymetrical shift of *Zoophycos* from the littoral–neritic to bathyal environments is synchronized with the tiering shift from shallow to deep (Figs 3 and 4c). The migration of *Zoophycos* is closely related to the increasing diversity of Phanerozoic marine invertebrates (Fig. 4a,c).

The shallow-tier in the neritic seas may be one of the most habitable environments for marine aerobic organisms. However, the macrofaunal competition in these places was drastically intensified with the increase of Phanerozoic marine invertebrate diversity. The disadvantaged organisms would have to adjust to other less habitable environments. As mentioned above, through three migrations from the neritic to bathyal beginning in the late Silurian, Famennian and Middle Triassic, respectively, Mesozoic *Zoophycos*-producers initially attempted the migration and adaptation to the slope environments. Further, Cenozoic *Zoophycos*-producers completely migrated, adapted to the bathyal environments. This is how the *Zoophycos*-producers were able to survive the ‘big five’ mass extinctions and stand as a major component of modern deep-sea biogenic traces^9^. The progressive increase in biological productivity since Cambrian and the greatly enhanced benthic nutrient level by virtue of post-Palaeozoic plankton blooms^34^,^35^ also played key roles in the tiering and bathymetrical evolution of *Zoophycos* (Figs 3 and 4).

Specifically, the Cambrian explosion and the substrate revolution from Neoproterozoic microbial mats to Cambrian mixed layers^36^ were established as the bio-environmental background for the origination of *Zoophycos*. Marine biodiversity was significantly increased after the Cambrian–Ordovician radiation. Plants^29^,^30^, acritarch blooms^37^, the second global oxygenic event^38^ (Fig. 4d–g), and the Middle Palaeozoic Revolution event (predators) in the Devonian^39^, improved the oxygen content and food supply in the neritic seas, resulting in the *Zoophycos* occurrence frequencies peak in the neritic sea of Devonian. However, the marine benthic communities and food chains were profoundly impacted by the Frasnian–Famennian and Devonian–Carboniferous transitional mass extinctions. The niches for neritic *Zoophycos* were restrained by the Carboniferous–Permian radiation, and the *Zoophycos* occurrence frequencies reduced further. Great changes took place in marine ecosystems at the Palaeozoic–Mesozoic transition^16^,^17^, among which was the increased competition in the neritic seas. The food supply and oxygen content in the bathyal and abyssal substrates was substantially increased by the phytoplankton blooms^35^ (Fig. 4e) from the Triassic to Middle Jurassic, when *Zoophycos* began to migrate to deeper-water environments. The nutrient level of deep-sea substrates was further improved by phytoplankton blooms^35^ and OAEs (Ocean Anoxic events)^40^, and *Zoophycos* completed the migration to the deep tier of the bathyal environments in the Cretaceous–Cenozoic.

In summary, the tiering and bathymetrical shifts of Phanerozoic *Zoophycos* reveal the adaptive radiation of the *Zoophycos*-producers in unsuitable environments. The Phanerozoic marine diversity expansion led to increased shallow tier competition in the neritic seas and forced disadvantaged organisms to adapt and migrate to less habitable bathyal–abyssal environments. With the deep-sea nutrient conditions greatly improved in the Mesozoic–Cenozoic, *Zoophycos* finally settled and prospered in the deep tier of bathyal seas in the Cretaceous–Cenozoic. It is also indicated that the physiological traits of the *Zoophycos*-producers evolved from aerobic in the Palaeozoic to a diverse ethological spectrum in the Cenozoic.

### Relationship between morphological macroevolution of *Zoophycos* and biological evolution

The morphology of *Zoophycos* demonstrates a three-stage evolution: from small, simple, spiraling, cocktail, circular/elliptical spreiten in one or two whorls without lobes in the Palaeozoic (e.g., *Zoophycos caudagalli*, *Zoophycos velum*); middle-sized, spiraling gently lobed circular/elliptical spreiten in three or five whorls with a few lobes in the Mesozoic (e.g., *Zoophycos* morphotype D^1^); to large, complex, spiraling, highly lobate spreiten in several whorls with numerous lobes in the Cenozoic (e.g., *Zoophycos rhodensis*, *Zoophycos insignis*) (Table 1 and Figs 1, 2, 3 and 4c). The initial stage of the complex lobate spreiten (*Zoophycos rhodensis*) may look like some branching forms of Mesozoic *Zoophycos*, which developed into a sub-circular, helical form with a central disk^41^. The evolution of this sub-circular form from the branching species might be a phylogenetic improvement in feeding by a ‘palingenetic’ shift in the behavioural sequence, and also indicate the trace-makers revert to a proven ancestral technique to improve foraging^42^,^43^. Palaeozoic *Zoophycos caudagalli* and Mesozoic–Cenozoic *Zoophycos insignis* both started with an initial U-tube or J-tube, and developed into sub-circular or lobate patterns. Feeding, gardening and farming in this proposed phylogenetic sequence are more a continuous track of improving efficiency rather than simple, discrete behavioral patterns. It culminated in forms of *Zoophycos insignis* that retained the initial U-tube or J-tube and inserted additional lobate whorls above the typical, sub-circular spreiten fields^41^,^44^. Cenozoic *Zoophycos rhodensis* was the inclusion of several, slender, lobate spreiten extended from the apex and separated by shorter elongated lobes^4^. This development must have been at least a trade-off in efficiency that utilized energy to create new lobes, but provided shorter access back to the surface^41^.

The macroevolution of *Zoophycos* is evident based on the above discussion of classic morphologies of *Zoophycos* in the Phanerozoic. The morphological changes of *Zoophycos* from simple to complex and from rough spreite structures to exquisite burrow systems reflect the strategic evolution (in considering the energy budget or efficiency) in feeding and living of *Zoophycos*-producers, which implies that animal behaviour can be well subjected to the basic laws of biological evolution, the same as have been practiced for anatomic feature, the biosphere, and ecosystem. This has already been suggested by the Jurassic–Cretaceous evolution of *Zoophycos* in France^1^.

The morphological macroevolution of *Zoophycos* can be ascribed to 1) the evolution of genetic material and trace-making strategy, and 2) the behavioural and physiological adaptation to the environmental changes of the *Zoophycos*-producers. The most important bio-environmental impact on the *Zoophycos*-producers is the increased competition in the habitable neritic seas due to Phanerozoic biodiversity expansion, which forces the *Zoophycos*-producers to migrate from shallow-tier in the neritic sea to deep-tier in the deep sea, along with enhanced ability to tolerate dysaerobic conditions, as exemplified by increasingly low oxygen content in slope to abyssal environments. Since the Mesozoic, especially since the Middle Jurassic, deep-sea bottom nutrient conditions were greatly improved by increased particulate organic carbon and dissolved organic carbon derived from surface plankton blooms^35^. The submarine nutrient and oxygen levels are commonly influenced by seasonal or other periodic factors^31^,^35^,^45^, resulting in the opportunistic colonization of the deep-sea substrates by the *Zoophycos*-producers. Therefore, environments select, eliminate, and transform organisms, and organisms also select (r- and K- selections), adapt and change environments, manifesting the coevolution of organisms and environments in the Phanerozoic.

### Probable *Zoophycos* producers

According to its general morphology, *Zoophycos* is usually considered to be produced by worm-like animals, of which several candidates have been proposed: sipunculida^1^,^2^, echiurida^46^, and polychaeta^11^,^47^. Based on the previous studies^1^,^23^,^24^,^46^ and our materials (Table 1 and Figs 2 and 4), the gradually increased width without much change in thickness in a single spreite, the alternated structure of the secondary lamellae^1^, and the sorted materials inside the laminae^23^, all suggest an animal capable of peristaltic motion incorporating several behavioural strategies (feeding, gardening, resting, excreting and so on). The diameter of the marginal tube is rather stable through the geologic time, which might indicate small worm-like producers that grow more in length than width. The stratigraphic record of *Zoophycos* through the past 500 Myrs does not seem be punctuated by any recognized major mass extinction events but reveals substantial bathymetric shift (Figs 2 and 4), which imply that the trace may have been produced by either a long-ranging conservative taxonomic group or several groups with similar behaviors and lifestyles that escaped mass extinctions.

## Methods

Through detailed analysis of 180 *Zoophycos* specimens and 291 papers on *Zoophycos* from 1821 to 2015, 448 valid points were obtained from the Cambrian to Quaternary (Table S1). Some considerations in building the *Zoophycos* database are listed below.

- Some reports of *Zoophycos* before the 1970s are not correct according to the present identification criteria. The Phanerozoic *Zoophycos* points we employed are derived only from papers published after 1970s with identifiable plates or from international palaeontological databases (e.g., Paleobiology Database). We only select specimens or records that follow the principal morphological constructs we recommended in the text, which allow safe assignment to *Zoophycos*. *Zoophycos* have different morphologies on bedding surface, in cross-section and three dimensions, so we use different descriptive terms for the three perspectives.
- The geologic age of *Zoophycos* points are mostly Stage-scaled, which are excerpted from the ‘geological setting’ sections in corresponding papers or related regional geological documents. For example, the paper reporting *Zoophycos* occurrence in the lower part of the Witteberg Formation in South Africa didn’t give the Stage for this Formation. By consulting related papers, a Givetian age was acquired.
- Marine environments (Fig. 4c) in recording the bathymetrical distribution of *Zoophycos* are largely divided into foreshore, nearshore, offshore, slope and abyssal according to the modern marine environmental zonation. The barrier island, lagoon or delta environments were subscribed into the above five marine zones according to water energy.
- In order to check the distribution of *Zoophycos* in correlation with temperature, latitude, climate and other factors, palaeolatitudinal data of *Zoophycos* points were transformed from the modern longitudes and latitudes using the software PointTracker. The modern longitudes and latitudes were mainly extracted from the corresponding literature. If this is not available, we positioned the occurrence area in Google Earth to obtain the longitudes and latitudes.
- Each point in the Phanerozoic *Zoophycos* database represents one occurrence, and we calculated the occurrences in each age and produced the *Zoophycos* occurrence frequency curve (Fig. 4b). The diameter of marginal tube, the dimension of spreiten and the number of whorls are all obtained from the original figures and plates in the references.

## Additional Information

**How to cite this article**: Zhang, L.-J. *et al.* *Zoophycos* macroevolution since 541 Ma. *Sci. Rep.* **5**, 14954; doi: 10.1038/srep14954 (2015).

## Supplementary Material

Supplementary Table S1

## Figures and Tables

**Table 1:** Statistics on diameter of the marginal tube, tier depth and number of whorls of the Phanerozoic Zoophycos*.

Age		Average diameter of the marginal tube(mm)	Average tier depth (cm)	Average number of whorls	Average width of spreite(cm)	Average total height of the spreite(cm)
Cenozoic	Palaeogene–Quaternary	4.06~4.69/74(135)	21.94~41.74/45(135)	11.08/24(135)	43.23/44(135)	26.34/25(135)
Mesozoic	Cretaceous	3.277~5.49/62(110)	5.25/14(110)	5.69/13(110)	36.35/32(110)	5.25/14(110)
	Triassic–Jurassic	2.57~4.39/28(58)	2.65/17(58)	2.50/20(58)	40.64/24(58)	2.51/16(58)
Palaeozoic	Carboniferous–Permian	3.72~4.20/42(75)	1.57/19(75)	2.00/32(75)	21.32/35(75)	1.72/17(75)
	Cambrian–Devonian	2.43~4.03/30(70)	0.38/12(70)	1.03/27(70)	17.98/33(70)	0.34/12(70)

^*^Recording style: e.g. 2.43~4.03/30(70) represents 70 points in the Cambrian–Devonian, including 30 valuable points (in getting the diameter of the marginal tube), and 2.43~4.03 is the average of the 30 points.
