# Supplementary material for: Zoophycos macroevolution since 541 Ma
Source: Sci Rep. 2015 Oct 9;5:14954. doi: 10.1038/srep14954 (PMC4598866; doi:10.1038/srep14954)
Supplement: Supplementary Table S1 [file srep14954-s1.pdf]

Supplementary Materials for

Zoophycos macroevolution since 541 Ma

Li-Jun Zhang, Ruo-Ying Fan, Yi-Ming Gong*

*To whom correspondence should be addressed. E-mail:

ymgong@cug.edu.cn

This PDF file includes:

Table S1

References

Table S1. Phanerozoic *Zoophycos* database

No. = Number of the item; **Basic morphology (3/S/H**), 3/S/H means the 3-D morphology (3), morphology in cross-section (S), and horizontal morphology (H), respectively. **W of MT (mm)** = Width of the marginal tube; **H of burrow (cm)**= Height of the burrow system, which is the distance between the top and bottom of the burrow system; **N of whorls** = Number of whorls; **Di of spreiten** = Dimension of spreiten, a×b×c = a cm in length, b cm in width and c cm in thickness, a and b are the maximum values of the whorls.; **Ref.** = references; **PD** in **Ref**. means Paleobiology Database (www.paleodb.org), **OC** in **REF**. means Our Collection. Empty box means the considering data was not found in the corresponding references.

No.	Era	Period	Epoch	Age	Locality	Longtitude/ latitude	Palaeo-latitude	Basic morphology(3/S/H)	W of MT (mm)	H of burrow (cm)	N of whorls	Di of spreiten （cm）	Host sediment	Environment	Ref.
1	**Cenozoic**	**Quaternary**	**Holocene**		Ontong Java Plateau, Micronesia	S 1 °37.5', E 159 °14.1'	-1.625	Cocks-tail shaped spreiten (H)		35			Calcareous nannofossil ooze	Bathyal zone	1
2					SONNE-114 Cruise Site 405	N 14 °40', E 119 °35'	14.67	Helicoidal *Spirophyton*-like spreiten (3)	2	13–27	14		Mud	Continental slope	2
3					SONNE-95 Cruise GIK17925-3	N 19 °51.2', E 119 °2.8'	19.85	Helicoidal *Spirophyton*-like spreiten (3)	2	33	37		Mud	Continental slope	3
4					LOMROG09-PC05, Lomonsov ridge	N 88.70 °, E 158.51 °	88.7	Helicoidal *Spirophyton*-like spreiten (3)		8	6		Mud	Continental slope	4,5
5					LOMROG09-PC10, Lomonsov ridge	N 89.45 °, W 130.38 °	89.45	Planar spreite (S)					Mud	Continental slope	4,5
6					AO96-14GC	N 89.45 °, W 130.38 °	87.01	Planar spreite (S)					Mud	Continental slope	4,5
7					M39029-4, M39029-8, Southern Iberian continental slope, Portugal	N 36 °02.5', W 8 °13.8'	36.042	Planar spreite (S)	2–3	30	4		homogeneously bioturbated unlithified sediments	Continental slope	6-10
8					70KL，Arabian sea, Indian Ocean	N 17 °30', E 61 °30'	17.5	Planar spreite (S)	5–15				mud	Bathyal zone	11
9					64KL，Arabian sea, Indian Ocean	N 19 °04', E 64 °41'	19.006	Planar spreite (S)	5–15				Mud	Bathyal zone	11
10					57KL，Arabian sea, Indian Ocean	N 20 °57', E 63 °07'	20.96	Planar spreite (S)	5–15				Mud	Bathyal zone	11
11					IMAGES core MD012388, Celebes Sea	N 6 °43', E 122 °56'	6.72	Planar spreite (S)					Nanofossil oozes and ash layers	Bathyal zone	12
12					SONNE 95 crusie, GIK17925-3, South China Sea	N 19 °51', E 119 °2.8'	19.83	Planar spreite (S)	4				Mud	Continental slope	13
13					METEOR cruis M39/1, core M39036, Atlantic	N 37 °48', W 9 °40.8'	37.8	Planar spreite (S)	4				Mud and clayey silts	Bathyal zone	14
14					METEOR cruis M39/1, core M39058, Atlantic	N 39 °2.4', W 10 °40.8'	39.006	Planar spreite (S)	4				Mud and clayey silts	Bathyal zone	14
15					METEOR cruis M39/1, core M39064, Atlantic	N 39 °59.2', W 9 °47.7'	39.99	Planar spreite (S)	4				Mud and clayey silts	Bathyal zone	14

16	**Cenozoic**	**Quaternary**	**Holocene**		Off NW Africa	N 13°53',<br>W 18°19’	13.88	Planar spreite (S)					Clay	Bathyal zone	15
17					Scotian slope, eastern Canada	N 42°40',<br>W 61°40'	42.66	Planar spreite (S)	4				Turbidite and debris flow deposits	Continental slope	16
18			**Pleistocene**	Middle	Sonne 187-61, South China Sea	N 11°25.5',<br>E 111°17'	11.425	Planar spreite (S)	4	20–28			Mudstone	Bathyal zone	17
19				Middle	Site 504, DSDP, Pacific	N 1°20',<br>W 83°46'	1.33	Planar spreite (S)					Clay	Bathyal zone	18
20				Gelasian	Site 73, DSDP, Pacific	S 2°14',<br>W 138°01'	-2.23	Planar spreite (S)	4				Mud	Continental slope	19
21					Off Valparaiso, Chile	S 33°01',<br>W 71°41'	-33.001	Planar spreite (S)					Ooze	Continental slope	20
22		**Neogene-Quaternary**	**Pliocene-Pleistocene**		Site 301, 309, Atlantic	N 27°01',<br>W14°20'	26.99	Planar spreite (S)	4	20–110				Continental slope	21,22
23					Site 310, 327,379, Atlantic	N 23°22',<br>W18°01	23.39	Planar spreite (S)	4					Continental slope	21
24					Site 329, Atlantic	N 19°01',<br>W19°59	19.05	Planar spreite (S)						Continental slope	21
25					Site 289,291, Atlantic	N 18°01',<br>W18°02'	18.03	Planar spreite (S)						Continental slope	21
26					Site 205, 337, 347, Atlantic	N 16°02',<br>W18°02'	16.05	Planar spreite (S)						Continental slope	21
27					Site 209, Atlantic	N 12°51',<br>W20°16'	12.89	Planar spreite (S)						Continental slope	21
28					Site 239, Atlantic	N 14°02',<br>W18°22'	14.05	Planar spreite (S)	4					Continental slope	21
29					Site 211, Atlantic	N 13°50',<br>W18°24'	13.85	Tongue-like spreiten (3)	3	8	3			Continental slope	21
30					Site 219, Atlantic	N 12°22',<br>W17°55'	12.39	Planar spreite (S)						Continental slope	21
31					GIK 16396, Iceland-Faeroe Ridge, Atlantic	N 61°24',<br>W 12°13'	61.52	Planar spreite (S)	5				Clay	Continental slope	23,24
32				Piacenzian-Upper	Leg 178, site 1101, Pacific	S 64°23',<br>W 70°16'	-64.37						Silty clay	Bathyal zone	25

33	**Cenozoic**	**Neogene**	**Pliocene**	Piacenzian	Site 504, DSDP, Pacific	N 1°20',<br>W 83°46'	1.32	Planar spreite (S)					Clay	Bathyal zone	18
34				Piacenzian	Kolymbia, Rhodes, Greece	N 36°15',<br>E 28°10'	36.25	Spiraling sheet-like lobate spreiten (3)	5	21	5	75×70×c	Limestone	Bathyal zone	26
35				Piacenzian	Leg 178, site 1096， Pacific	S 67°34',<br>W 76°58'	-67.56						Silty clay	Bathyal zone	25
36				Piacenzian	DSDP 203core， Pacific	S 22°09',<br>W 117°32'	-22.016	Planar spreite (S)	3		9		Nannofossil ooze	Bathyal zone	27
37				Piacenzian	Punta Corda, Ecuador	N 0°55'23",<br>W79°46'33"	0.91		5	5	8		Siltstone–mudstone	shelf edge/uppermost slope	28
38				Piacenzian	Boso Peninsul, Japan	N 34°56',<br>E 139°50'	34.92	Helicoidal *Spirophyton*-like spreiten (3)	5	22	13	51.69×55.84	Turbidite	Middle bathyal	29-34
39				Zanclean-Piacenzian	Sulu sea, Core 14210-5	N 9°50',<br>E 119°48'	9.83	Planar spreite (S)		40			Mud and sand	Bathyal zone	35
40				Zanclean-Piacenzian	Sulu sea, Core 14224-1	N 7°40',<br>E 121°30'	7.67	Planar spreite (S)					Mud and sand	Abyssal zone	35
41				Zanclean-Piacenzian	Sulu sea, Core 14233-5	N 8°52',<br>E 121°52'	8.87	Planar spreite (S)					Mud and sand	Abyssal zone	35
42				Zanclean	ANDRILL 1B, Antarctic	S 77°46',<br>E 171°28'	-77.76	Planar spreite (S)	2–4	2–6	4		Mud	Continental slope	36
43				Zanclean	Ecuador	N 1.0°,<br>W 79.8°	0.8						Green, tuffaceous mudstone	Bathyal zone	37, PD
44			**Miocene**	Messinian-Piacenzian	Leg 178, site 1095, Pacific	S 66°59',<br>W 78°29'	-67.05						Silty clay	Bathyal zone	25
45				Messinian	North Taranaki coast, New Zealand	S 39°02',<br>E 174°11'	-44.36	Spiraling sheet-like spreiten (3)		26	4	13.16×9.04	Mudstone and silty sandstone	Bathyal zone	38
46				Messinian	estern Raukumara Peninsula, New Zealand	S 38°50',<br>E 178°30'	-38.82						Sandstone	Upper bathyal zone	39
47				Messinian	Leg 119, site 746, Indian Ocean	S 56°33',<br>E 82°52'	-56.07						Clay, silt clay	Abyssal zone	40
48				Messinian	Rangamati-Chittagong Road section, Bangladesh	N22°34'05.59",<br>E 91°56'13.31"	16.61						Mudstone	shelf	41
49				Messinian-Zanclean	Punta Perro, Chile	S 33°54'52.12",<br>W 71°50'38.75"	-34.27	Planar spreite (S)					Sandstone	Continental slope	42

50	**Cenozoic**	**Neogene**	**Miocene**	Tortonian-Messinian	DSDP 223core, Indian Ocean	S 2°48', E 87°36'	-7.49	Planar spreite (S)	5				Nanofossil ooze	Bathyal zone	27
51				Tortonian-Messinian	Site 504, DSDP, Pacific	N 1°20', W 83°46'	0.41	Planar spreite (S)					Interbedded limestone, chalk, and chert	Bathyal zone	18
52				Tortonian-Messinian	Bardados ridge, leg 78, DSDP, Atlantic	N 15°22', W59°20'	15.23		4	8	7		Mud	Bathyal zone	43
53				Tortonian?	Site 178, DSDP, Pacific	N 56°32', W 146°30'	52.87	Planar spreite (S)	4				Mud	Bathyal zone	19
54				Tortonian	Coniale, Italy	N 44°15', E 11°46'	43.96	Planar spreite (S)	6			40×15	Mudstone	Bathyal zone	44
55				Tortonian	Punta Litre, Chile	S 37°11.73', W73°33.64'	-37.58						Sandstone–mudstone	Bathyal zone	45
56				Serravallian-Tortonian	Monteleon Di puglia, faeto, Italy	N 41°18', E 15°09'	40.98	Spiraling lobate spreiten (3)	5			20×24×c	Limestone–marl	Bathyal zone	46
57				Langhian-Serravallian	Adana, Turkey	N 37°00', E 35°10'	36.72	*Rhizocorallium*-like spreiten (3)	4		6	10×7×c	Sandstone	Abyssal zone	47
58				Langhian-Serravallian	North Adana, Turkey	N 37°06', E 35°23'	36.72	U- or J-form spreiten (H)	5			1×5×c	Sandstone	Slope–Abyssal zone	48
59				Langhian-Serravallian	Carriacoa, Grenadines, Lesser Antilles	N 15°10', W 62°30'	15.13	Helicoidal spreiten (S)				a×13.7×c	Sandstone	Outer island shelf	49
60				Langhian	Baden, Vienna Basin, Austria	N 47°59', E 16°13'	47.64	Planar spreite (S)	4				Clay	Upper offshore	50
61				Langhian	Baden-Sooss, Austria	N47°59.00', E 16°12.84'	47.65						Marl and shale	Bathyal zone	51
62				Langhian-Messinian	Monte dei Corvi, Conera Riviera, Ancona, Italy	N 43°51', E 7°50'	43.58		5–10	50			Limestone	Continental slope	52
63				Langhian	Grund, Austria	N 48°38', E 16°03'	48.95	J-form helicoidal lobate spreiten with long, *Rhizocorallium*-like lobes (3)	2–3	35	3-5	40×50×70	Sandstone	Upper offshore	53,54
64				Aquitanian	Cabo San Pablo beds, Fuegian Andes, Agentina	S 54°30', W 66°40'	-55.17	Helicoidal circular spreiten (3)					Mudstone	Continental slope	55
65				Aquitanian	Desdémona Formation, Fuegian Andes, Agentina	S 54°30', W 66°40'	-55.17	Helicoidal lobate spreiten (3)					Mudstone	Continental slope	55
66				Aquitanian	Leg28, site 269, Pacific	S 61°41', E 140°04'	-62.3						Clay, silt clay	Abyssal zone	56,57

67	**Cenozoic**	**Neogene**	**Miocene**	Aquitanian	DSDP 192core, Pacific	N 53 °60', E 164 °42'	48.48	Spiraling flat spreiten (H)					Limestone	Bathyal zone	27
68				Aquitanian-Messinina?	DSDP 223 core, Indian Ocean	S 2 °48', E 87 °36'	-3.31	Planar spreite (S)	4		6		Marly chalk	Bathyal zone	58
69				Aquitanian?	Caribbean cores 153, Caribbean sea	N 13 °40', W72 °27'	13.44	Planar spreite (S)	3				Clay	Bathyal zone	59
70				Aquitanian-Burdigalian	Leg 66 core, Pacific	N 14 °56', W94 °49'	15.52	Planar spreite (S)	4				Clay	Continental slope	60
71		**Paleogene**	**Oligocene**	Chattian	Leg28, site 267, Pacific	S 59 °16', E 104 °29'	-58.59						Nanofossil ooze, chalk	Abyssal zone	56,57
72				Chattian	Leg 113, site 693, Pacific	S 70 °50', W 114 °34'	-72.01						Nannofossil ooze, mudstone	Bathyal zone	61
73				Rupelian	Eastancia Mar ía Cristina beds, Fuegian Andees, Agentina	S 54 °30', W 66 °40'	-56.12	Planar spreite (S)					Mudstone	Continental slope	55
74				Rupelian	DSDP 206core, Pacific	S 32 °00', E 165 °15'	-45.3	Planar spreite (S)	5				Nannofossil ooze	Bathyal–abyssal zone	27
75				Rupelian	Oldenburg,northwestern Deustchlands, German	N53 °12', E 8 °22'	52.74	Coiled spreiten (H)	4				Clay	Continental slope	62
76				Rupelian	Oldenburg,northwestern Deustchlands, German	N53 °12', E 8 °22'	52.74	Spiraling tongue-shaped spreiten (3)	4				Clay	Continental slope	62
77				Rupelian	Stern Creek, South Nelson, New Zealand	S 41 °17', E 173 °13'	-52.33	Helicoidal spiral spreiten (3)				66 ×64 ×c	Mudstone, marl and limestone	Continental slope	63
78				Rupelian	Motunau River, North Canterbury, New Zealand	S 42 °52', E 173 °00'	35.79	Helicoidal loabte spreiten	4			72 ×74 ×c	Limestone	Lower offshore	64
79				Rupetian	Hsuehshan Range, Taiwan	N24 °31.99', E121°23.77’	24.53	J-form spiraling spreiten (H)				48 ×40 ×c	Sandstone	Lower offshore	65
80				Rupelian	Zone IPM4 section Wujskie A, Poland	N49 °33.19', E 22°18.01’	49.44	Planar spreite (S)	2				Limestone	Bathyal zone	66
81				Rupelian-Chattian	Maritime ALPS, protula septaria, Italy	N 44 °15', E 7 °10'	38.42	U-form spiraling spreiten (H)	4			12 ×24 ×c	Siltstone and mudstone	Bathyal zone	67
82			**Eocene**	Priabonian-Messinian	Leg 113, site 689, Atlantic	S 64 °31', E 03 °06'	-63.31						Diatom-rich nannofossil ooze	Continental slope	61
83				Priabonian	DSDP 161core, Pacific	N 10 °14', W 139 °57'	2.54	Planar spreite (S)	4				Radiolarian ooze	Continental slope	27

84	Cenozoic	Paleogene	Eocene	Priabonian	Afales Bay, northern Ithaca, Ionian Isles, Greece	N 38 °10', E 20 °31'	32.79	Helicoidal lobate spreiten (3)	6	23	5	40×95×20		Bathyal zone	68
85				Priabonian	Afales Bay, northern Ithaca, Ionian Isles, Greece	N 38 °10', E 20 °31'	32.79	Helicoidal lobate spreiten (3)	5		2	60×63×C		Bathyal zone	68
86				Priabonian	Site 161A, DSDP, Pacific	N 11 °49', W 139 °12'	4.18	Planar spreite (S)	4					Bathyal zone	19
87				Priabonian	Site 41, DSDP, Pacific	N 20 °01', W 140 °22'	12.25	Planar spreite (S)					Nannofossil ooze	Bathyal zone	69
88				Priabonian	North Dalmatia, Croatia	N 43 °34', E 16 °48'	38.38	Coiled spreiten (H)				6×8×c	Sandstone	Offshore zone	70
89				Priabonian	Garulla-Amandola road, Italy	N 42 °59', E 13 °18'	38.02	J-form spiraling spreiten (3)	4			60 ×80×c	Siltstone	Bathyal–Abyssal zone	71
90				Bartonian	Camerino, Italy	N 43 °08', E 13 °04'	38.07	U-form spiraling spreiten (H)					Limestone	Continental slope	72
91				Bartonian-Priabonian?	Site 605, North American continental margin off New Jersey, USA	N 40 °25', W 72 °45'	39.83		5	9	19		Mudstone	Continental slope	73
92				Bartonian	Villapiana, Italy	N 29 °50', E 16 °27'	24.7	Spirally coiled spreiten (H)				5 ×7×c	Shale	Bathyal–Abyssal zone	74
93				Bartonian-Priabonian	Cerro Colorado Formation, Fuegian Andes, Agentian	S 54 °30', W 66 °40'	-56.79	U-form spiraling spreiten (H)					Mudstone	Continental slope	75
94				Bartonian-Langhian	Leg 113, set 696, Atlantic	S 61 °51', W 42 °56'	-61.72						Mudstone	Continental slope	61
95				Bartonian-Chattian	Leg 119, site 737, Pacific	S 50 °14', E 104 °29'	-50.2						Calcareous claystone	Continental slope	40
96				Bartonian	Villapiana, Itally	N39 °51'41.03", E 16 °29'30.02"	34.72	Helicoidal spreiten (H)	5			8×10×c	Carbonate and turbidites	Continental slope	74
97				Bartonian	Teherau Valley section, Buzau, Romania	N45 °22'50.57", E26 °27'57.44"	43.97	Helidoidal spreiten (H)				36 ×36 ×c	Sandstone	Bathyal zone	76
98				Lutetian	W of Jaca, southern Pyrenees, Spain	N 42 °34', W 0 °35'	40.72	U-form spiraling spreiten (3)	4			10×80×c	Sandstone	Bathyal zone	77
99				Lutetian-Bartonian	DSDP 161core, Pacific	N 10 °14', W 139 °57'	2.45	Planar spreite (S)	5				Radiolarian ooze	Continental slope	27
100				Lutetian	Slopnice, Poland	N 49 °22', E 20 °20'	38.47	U-form spiraling spreiten (3)	4	5		18×20×5	Siltstone	Continental slope	OC

101	Cenozoic	Paleogene	Eocene	Ypresian-Bartonian	Whaingaroan, Waipara district, North Caterbury, New Zealand	S 43°03', E 172°45'	-43.005	J-form spiraling spreiten (3)	5–7	2.5		26.5×12×c	Limestone	Lower offshore	78
102				Ypresian	Krościenko-Łakcica, Poland	N 49.46°, E 20.37°	40.69						Sandstone and mudstone	Bathyal zone	79
103				Ypresian?	Punta, Fuegian Andes, Agentina	S 54°30', W 66° 40'	-56.97	Simple, circular spreiten (H)					Sandstone and mucstone	Bathyal zone	75
104				Ypresian	Agost section, Betic Cordillera, Spain	N 38°26'23.84", W 0°37'48.17"	34.13	U-shaped spiral spreiten	5			50×60×c	Marly limestone	Bathyal zone	80
105				Ypresian-Bartonian?	Carpathians, Bzova, Czechoslovak	N49°22', E 18°15'	43.66	J-shaped spiral spreiten (3)	4	8	5	20×34×c	Fine sandstone	Continental slope	81-84
106				Ypresian	Site 41, DSDP, Pacific	N 20°01', W 140°22'	10.53	Planar spreite (S)					Nanofossil ooze	Bathyal zone	69
107				Bartonian-Rupelian	Gönen Kale hill, Turkey	N 39°39.16', E 27°52.31’	39.69	Fan-shaped spreiten (H)				19×10×c	Calcarenite	Outer shelf	85
108				Ypresian-Lutetian	Reno Pass section, Illam, Zagros Basin, Iran	N 33°42.27', E 46°24.89’	20.83						Shale and wackestone	Bathyal zone	86
109				Ypresian-Lutetian	Hajbakhtiar section, Zagros Basin, Iran	N 33°48.07', E 46°8.29’	20.95						Shale and wackestone	Bathyal zone	86
110					Montanare section, Trasimeno area, Italy	N 43°4.36', E 12°14.85’	35.09	U-form spiraling spreiten (H)	6			a×30×c	Bioclastic mud	Bathyal zone	87
111				Ypresian?	Fuegian Andes, Argentina	S 54°30.76', W 66°18.1’	-56.99	Helicoidal spreiten (3)	5	5	4		Mudstone	Continental slope	88
112				Thanetian-Ypresian	Anhovo (H), Italy	N 46°04'16", E 13°36'22"	35.97	Tongue-like spreiten (H)	4			30×20×c	Limestone	Bathyal zone	89
113				Thanetian-Ypresian	Anhovo (H), Italy	N 46°04'16", E 13°36'22"	35.97	Plannar spreite (S)	4			75×75×c	Limestone	Bathyal zone	89
114				Thanetian-Ypresian	Pedrosa (M), Italy	N 46°09'50", E 13°29'52"	36.07	Planar spreite (S)	4		8	2×2×c	Limestone	Bathyal zone	89
115			Paleocene	Thanetian	Zumaya section, Itzurun beach, Spain	N 43°17'57", W 2°16'07"	37.43	Spiraling circular spreiten (H)				35×35×c	Muddy limestone	Bathyal zone	90-92
116				Thanetian	Zumaya section, Itzurun beach, Spain	N 43°17'57", W 2°16'07"	37.43	Tongue-like spreiten (H)	3–5			5×25×c	Muddy limestone	Bathyal zone	90-92
117				Thanetian	Zumaya section, Itzurun beach, Spain	N 43°17'57", W 2°16'07"	37.43	Flower-shaped spreiten (H)				6×8×c	Muddy limestone	Bathyal zone	90-92

118	**Cenozoic**	**Paleogene**	**Paleocene**	Thanetian	Zumaya section, Itzurun beach, Spain	N 43°17'57", W 2°16'07"	37.43	Spiral spreiten (H)				140×140×c	Muddy limestone	Bathyal zone	90-92
119				Thanetian	Zumaya section, Itzurun beach, Spain	N 43°17'57", W 2°16'07"	37.43	Conical shaped spreite (S)		30			Muddy limestone	Bathyal zone	90-92
120				Selandian	Sopelana section, Basque Basin, northern Spain	N 43°23.26', W 2°59.691'	37.55	Spiraling lobate spreiten (H)	2–2.5				Grey marls	Bathyal zone	93
121				Danian	Busche village, Feltre, Italy	N 46°00', E 11°54'	41.11	Spiraling sheet-like spreiten (H)	5			40×60×c	Limestone	Bathyal zone	94-96
122				Danian	Ponte nelle Alpi, Italy	N 46°08', E 12°13'	41.1	Spiraling sheet-like spreiten (H)	5			20×100×c	Limestone	Bathyal zone	94,95
123				Danian	Rio de la vega, Tarifa, Spain	N 36°00', W 5°36'	31.81	U-form spiraling spreiten (H)	4		2	8×20×c	Calcareous turbidite	Bathyal zone	97
124				Danian	Cadiz, Spain	N 36°31', W 6°17'	32.34	Helicoidal conical spreiten (H)	4			7×12×c	Calcareous turbidite	Bathyal zone	97
125				Danian	Andalucia, Spain	N 36°30', W 6°16'	32.33	U-form spiraling spreiten (H)	5		4	10×15×c	Calcareous turbidite	Bathyal zone	97
126				Danian	Caravaca section, Spain	N 38°03'47", W 1°51'57"	32.18	U-form spiraling spreiten (H)	6			5×7×c	Marly limestone	Continental slope	98
127				Danian	Leg 74, Walris Ridge, DSDP, Atlantic	S 29°30', E 3°40'	-37.25	Planar spreite (S)	2–3				Chalk	Abyssal zone	99
128				Danian-Selandian	Carpathians, Val. Klobouky, Czechoslovak	N49°09', E 18°00'	43.44	J-form spiraling spreiten (H)	2–3			8×20×c	Marlstone	Continental slope	81-84
129				Danian-Selandian	Qpquqwe River and Te Kau Kau Point, south-estern, Wairarapa, New Zealand	S 41°16', E 175°19'	-54.47	Planar spreite (S)	5				Limestone	Bathyal zone	63
130				Danian	Kožbana, Goriška Brda, Slovenia	N 46°02', E 13°32'	35.9	Helicoidal spreiten (H)				60×60×c	Marl	Bathyal zone	100
131				Danian-Selandian	Pahoa River, Southeast Wellington, New Zealand	S 41°20', E 174°49'	-54.75	Planar spreite (S), helicoidal spreiten (3)	4			180×160×c	Limestone	Bathyal zone	101
132				Danian	Deva, Spain	N 43°18', W 2°14'	37.42						Limestone	Bathyal zone	102
133				Danian?	Passo delle, Capannele, L'Aquila, Italy	N42°25', E 13°33'	32.36	Helicoidal lobate spreiten (H)	4			10×15×c	Calcareous marls	Bathyal zone	103
134				Danian	Caravaca de la Cruz, Murcia Province, Spain	N 38°06', W 1°51'	32.12	Helicoidal lobate spreiten (3)	4–6	90	7	65×65×90	Marly limestone	Bathyal zone	104,105

135			**Paleocene**	Danian	Uzgrůn, Moravia, Czech Republic	N 49°28',<br>E 18°24'	39.36	U-form spiraling spreiten (H)	2–4			6×9×c	Sandstone	Bathyal zone	[106]
136	**Mesozoic**	**Cretaceous**	**Late**		Livorno, Italy	N 42°32',<br>E 0°21'	30.96	Spiraling circular spreiten (3)	3				Limestone	Bathyal zone	[107]
137				Maastrichtian-Rupelian	North Canterbury and south Malbrough, New Zealand	S 43°19',<br>E 173°12'	-55.06	Spiraling tongue-like spreiten (3)	4–5		15	100×90×c	Limestone	Bathyal zone	[108,109]
138				Maastrichtian-Ypressian	Site 41, DSDP, Pacific	N 20°01',<br>W 140°22'	10.53	Planar spreite (S)						Bathyal zone	[69]
139				Masstrichtian-Danian	Bidart section, France	N 43°26'31",<br>W 1°35'33"	37.53	Spiraling tongue-like spreiten (3)	4			40×40×c	Cretaceous marlstone and Paleogene marly limestone	Bathyal zone	[110,111]
140				Masstrichtian-Danian	Sopelana, Spain	N 43°22'41",<br>W 3°00'59"	37.54	Planar spreite (S)	4				Cretaceous marlstone and Paleogene marly limestone	Bathyal zone	[111]
141				Masstrichtian	Neuberg, Austria	N 47.7°,<br>E 15.5°	35.6						Sandstone and silty sandstone	Coastal	[112], PD
142				Masstrichtian	New Jersey coastal Plain, USA	N 40°11',<br>W 74°15'	40.43	Planar spreite (S)	2–3				Sandstone	Offshore	[113]
143					Leg 119, site 738, Indian Ocean	S 62°43',<br>E 82°47'	-65.38						Limestone chalk	Bathyal zone	[40]
144				Masstrichtian	Caribbean cores 146, Caribbean sea	N 14°09',<br>W69°08'	13.54	Planar spreite (S)	3	8	14		Clay	Bathyal zone	[59]
145				Masstrichtian	Kulsti Rende, Stevens Klint, Denmark	N 55°57',<br>E 11°35'	48.01	Planar spreite (S)	2–4				Flint	Bathyal zone	[114]
146				Masstrichtian	Englebel, limburg, Netherlands	N 51°26',<br>E 6°04'	43.61	Helicoidal lobate spreiten (3)	2–4				Flint	Bathyal zone	[114]
147				Masstrichtian	Limhamn, Scania, Sweden	N 55°34',<br>E 12°55'	47.58	U-form spiraling lobate spreiten (3)	2–4		7		Flint	Bathyal zone	[114]
148				Masstrichtian	Kjølby Gaar, NW Jylland, Denmark	N 56°14',<br>E 10°36'	48.31	Planar spreite (S)	3–5		4		Flint	Bathyal zone	[58]
149				Masstrichtian	Dania Quarry, near Assens, Denmark	N 55°16',<br>E 9°53'	47.34	Planar spreite (S)	2–3			8×20×c	Marl–chalk	Bathyal zone	[115]
150				Masstrichtian	Tüna, Tibet, China	N 27°51',<br>E 89°04'	-24.34	Spiraling spreiten (H)	2–4			6×9×c	Limestone	Shelf margin	[116]
151				Masstrichtian	La Lajilla, Mexico	N 23°40',<br>W 98°45'	29.82	U-form spiraling spreiten (H)	2–3			8×14×c	Sandstone	Lower offshore	[117]

152	Mesozoic	Cretaceous	Late	Masstrichtian	Northern and central New Jersey, USA	N 40 °41', W 74 °14'	40.92	Spiraling elliptical spreiten (3)	10	20		15×20×c	Muddy sand	Offshore	113
153				Masstrichtian	Bottaccione Gorge at Gubbio, Italy	N 43 °22', E 12 °35'	31.4						Limestone	Bathyal zone	118
154				Masstrichtian	Agos, Spain	N 38 °29', E 0 °36'	38.49	U-form spiraling spreiten (H)	3–5			4×14×c	Limestone	Bathyal zone	119,120
155				Masstrichtian	Leg 35, site 323, Pacific	S 63 °41', W 97 °60'	-60.81						Claystone	Abyssal zone	121
156				Maasstrichtian-Danian	Leg 74, Walris Ridge, DSDP, Atlantic	S 29 °30', E 3 °40'	-40.15	Planar spreite (S)	2–3				Chalk	Abyssal zone	99
157				Masstrichtian	Lameta Ghat section, Jabalpur area, India	N 23 °8'07", E 79 °51'07"	-33.688	Irregularly thin menisci (H)	3–4				Limestone	Lagoon	122
158				Masstrichtian	Kožbana, Goriška Brda, Slovenia	N 46 °02', E 13 °31'	27.79	J-form spiraling spreiten (H)	1–2			60×70×c	Marl	Bathyal zone	100
159				Masstrichtian	San Diego, California, USA	N 32 °40', W117 °14'	30.82	broad, flat, essentially circular spreiten (H)	5–6				Mudstone	Bathyal zone	123
160				Masstrichtian	Mead Hill, New Zealand	S 42 °20', E 173 °43'	-75.24						Chert	Bathyal zone	124
161				Maastrichtian	Vagge, Crocefieschi, Italy	N 44 °34.01', E 8°57.01’	33.17	J-form spiraling spreiten with lobes (H)				a×17×c	Mixed siliciclastic–calcareous	Bathyal zone	125
162				Campanian	Leki quarry, Poland	N 49 °49.906', E 20°1.296’	41.79	Helicoidal lobate spreiten (H)		5		25×17.5×c	Sandstone	Bathyal zone	126
163				Campanian	Arras, cuifial, England	N 53 °52', W 0 °40	46.27						Chalk	Bathyal zone	127
164				Campanian	Warren farm, England	N 50 °48', W 1 °09'	43.26						Chalk	Bathyal zone	127
165				Campanian	St-Julien, France	N 48 °17', E 3 °51'	40.56						Chalk	Bathyal zone	127
166				Campanian	Arcis, France	N 48 °24', E 4 °13'	40.65						Chalk	Bathyal zone	127
167				Campanian	Leybach, Germany	N 47.4843 °, E 10.3053 °	32.97	U-form spiraling spreiten (H)	4–5		5	10×15×c	Marlstone	Bathyal zone	128
168				Campanian	Fachina Joch, Austria	N 47 °27`', E 9 °54'	33.02	Helicoidal spreiten (3)	4–5		5	10×14×c	Marlstone	Bathyal zone	128

169	Mesozoic	Cretaceous	Late	Campanian	Schmiedlaine Creek, Germany	N 47.6746°, E11.4427°	37.65	U-form spiraling spreiten (H)	4–5		4	11×13×c	Marlstone	Bathyal zone	[128]
170				Campanian	Western Alabama, USA	N 32°19', W 86°54'	35.7	Planar spreite (S)	5				Marl and limestone	Bathyal zone	[129,130]
171				Campanian	Spocari Quarry, western Alabama, USA	N 32°16'18", W 87°47'03"	35.86	Planar spreite (S)	4				Marl–chalk	Bathyal zone	[129,130]
172				Campanian	E1 chingue bluff, Chile	S 51°10', W 72°30'	-52.95	Planar spreite (S)	3–4				Sandstone	Bathyal zone	[131]
173				Santonian	Caribbean cores 153, Caribbean sea	N 13°40', W72°27'	8.07	Planar spreite (S)	2–3				Clay	Bathyal zone	[59]
174				Santonian	Bystrý potok section, Ostrava, Czeck	N 49°34', E 18°18'	39.472	Spiral spreiten (H)				4×6×c	Turbiditic sandstone and shale	Bathyal zone	[132]
175				Santonian to Early/Lower Campanian	Buen Suceso Bay, Argentina	S 54.8°, W 65.3°	-57.9						Sandstone	Offshore	[133], PD
176				Coniacian	Margate, England	N 51°22', E 1°26'	43.72						Chalk	Bathyal zone	[127]
177				Coniacian	Dieppe, France	N 49°55', E 1°05'	42.31						Chalk	Bathyal zone	[127]
178				Coniacian	Vitry, France	N 48°27', E 4°20'	40.68						Chalk	Bathyal zone	[127]
179				Coniacian	Butser Hill, England	N 51°01'12.13", W 0°58'49.18"	43.48						Flint	Bathyal zone	[134]
180				Coniacian	Beachy head, England	N 50°44'14.83", E 0°14'51.35"	43.15						Flint	Bathyal zone	[134]
181				Coniacian	western-central Kansas, USA	N 38°38', W 100°04'	43.55	U-form spiraling spreiten (H)	3–6			10×23×c	Chalky limestone	Lower offshore	[135]
182				Turonian-Coniacian, Santonian-Maastrichtian	Scotian shelf, Canada	N 43°44', W 61°09'	40.92						Limestone	Deep shelf zone	[136]
183				Turonian-Coniacian, Santonian-Maastrichtian	Grand banks, Canada	N 45°01', W 50°02'	39.88						Limestone	Deep shelf zone	[136]
184				Turonian-Campanian	Colorado, USA	N 40°34', W 103°56'	46.31	Planar spreite (S)	1–2				Limestone	Bathyal zone	[137]
185				Turonian-Campanian	Lyons Quarry section, Corolado,USA	N 39°42', W 106°21'	46.06	Planar spreite (S)	2–5	5			calcareous shale and marlstone	Bathyal zone	[137]

186	Mesozoic	Cretaceous	Late	Tuonian	Rzki, Poland	N 49 °49', E 19 °22'	41.77	U-form spiraling spreiten (3)	2–4	5	3	10×20×c	Marly limestone	Bathyal zone	OC
187				Tuonian	Black band, England	N 53 °36', W 0 °31'	46.03						Chalk	Bathyal zone	127
188				Tuonian	Sens bed well's columnar, France	N 48 °16', E 3 °56'	40.53						Chalk	Bathyal zone	127
189				Tuonian	Western Interior seaway of Alberta, Canada	N 52 °18', W 115 °47'	60.84						Mudstone	Shelf	138
190				Tuonian	Banos de la Hedionda, Betic Cordillera, Spain	N 36 °23'44", W 5 °15'42"	36.396	Planar spreite (S)	2–3	3			Marl limestone	Bathyal zone	139-141
191				Tuonian-Coniacian	Western Interior seaway of Alberta, Canada	N 52 °14', W 115 °20'	56.09	Planar spreite (S)	3–5				Sandstone	Upper offshore	142
192				Tuonian	Pembina Carrot Creet oilfield of west-central Alberta, Canda	N 52 °15', W 115 °37'	56,17	Vertical spiral spreiten (H)	8				Mudstone	Shelfal	143
193				Turonian	Western Interior Seaway, Canada	N 52 °46.4', W 116°50.18’	56.92						Siltstone and sandstone	Upper offshore	138
194				Turonian	Seebe, Albert, Canada	N 51 °05.04', W 115°3.74’	54.95	U-form spiraling spreiten (H)					Argillaceous siltstone	Upper offshore	144
195				Cenomanian	El Chorro, Penibetic, Spain	N 36 °54.87', W 4°45.92’	26.54	Planar spreite (S)	4				Marl limestone	Bathyal zone	141
196				Cenomanian-Turonian	Rybie section, Poland	N 49 °47.02', E 20°19.89’	40.23	Planar spreite (S)	2–3				Marlstone	Bathyal zone	145
197				Cenomanian	Sztolnia Section, Poland	N 49 °24.082', E 20°31.537’	33.27	Planar spreite (S)	1				Black shale	Bathyal zone	146
198				Cenomanian	Bottaccione section, Gubbio area, Italy	N 43 °21.701', E 12°34.309’	28.04	Planar spreite (S)	2–3				Black shale	Bathyal zone	147
199				Cenomanian	Contessa section, Gubbio area, Italy	N 43 °22.535', E 12 °33.646’	28.06	Planar spreite (S)	2–3				Black shale	Bathyal zone	147
200				Cenomanian-Zanclean	Leg 113, site 690, Atlantic	S 65 °09', E 01 °13'	-65.3						Nannofossil ooze	Bathyal zone	61
201				Cenomanian	Asham, Beachy head, England	N 50 °44', E 0 °17'	43.12						Chalk	Bathyal zone	127
202				Cenomanian	Antifer, France	N 49 °38', E 0 °10'	42.03						Chalk	Bathyal zone	127

203	**Mesozoic**	**Cretaceous**	**Late**	Cenomanian-Turonian	Devor, England	N 51 °07', E 1 °18'	41.29	Helicoidal sheet-like spreiten (3)	3–6	3		40×40×c	Chalk	Lower offshore	[148]
204				Cenomanian	Demoplis, Alabama, USA	N 32 °30', W 87 °50'	27.79	Planar spreite (S)	4–7	2		20×100×c	Marl and chalk	Bathyal zone	[149]
205				Cenomanian-Turonian	Northwestern James Ross island, Antactic	S 65 °10', W 58 °12'	-70.65	Planar spreite (S)	1.5				Mudstone	Bathyal zone	[150]
206				Albian, Santonian-Campanian, Maastrichtian	Fuegian Andes, Agentina	S 55 °14', W 67 °30'	46.12	Planar spreite (S)					Mudstone	Slope–volcaniclastic apron setting	[151]
207			**Early**	Albian	Le Brecce, 3km west of the town of Piobbico, Marche, Central Italy	N 43 °35'04", E 12 °29'12"	27.35	Planar spreite (S)	3–5				Shale	Bathyal zone	[152]
208				Albian	Calgary, Canada	N 51 °40', W 112 °09'	49.49	Planar spreite (S)	3–5				Muddy sandstone	Shelf	[153]
209				Aptian	Alexander island, Antarctica	S 70.8 °, E 68.5 °	-69.4						Sandstone and shale	Offshore	[154], PD
210				Aptian	Yuasa, Japan	N34 °02', E135 °10'	41.33	J-form spirally coiled spreiten (3)	3–4	3	2	6×8×3	Sandstone	Bathyal zone	[155]
211				Aptian-Coniacian	James Ross Island, Antarctic	S 64 °10', W 58 °20'	-67.42	Planar spreite (S)	2–3	3.5	3-7	8×7×c	Mudstone	Bathyal zone	[156]
212				Aptian-Cenomanian?	Vergons, France	N 43 °54', E 6 °35'	35.03		3–5			30×40×c	Marl–limestone alternation	Bathyal zone	[157-159]
213				Aptian-Cenomanian?	Angles, France	N 43 °56', E 6 °33'	35.04		3–5			30×40×c	Marl–limestone alternation	Bathyal zone	[157-159]
214				Aptian-Cenomanian?	Carajuan, France	N 43 °47', E 6 °26'	34.91		3–5			30×40×c	Marl–limestone alternation	Bathyal zone	[157-159]
215				Barremian	Pas de la Cluse, France	N 44 °27', E 5 °01'	34.33	Spiraling simple circular spreiten (H)	5			a×60×c	Calcarenitic turbidite	Bathyal zone	[160]
216				Barremian	Pas de la Cluse, France	N 44 °27', E 5 °01'	34.33	Spiraling lobate spreiten (H)	20–30	3		a×160×c	Calcarenitic turbidite	Bathyal zone	[160]
217				Barremian-Hauterivian	Alexander island, Antarctica	S 70.8 °, E 68.5 °	-65.6						Sandstone and shale	Offshore	[154], PD
218				Hauterivian	Vocontian Trough, La Charce, Marie, France	N 44 °31'12.48", E 5 °27'31.32"	34.49	J-form spiraling spreiten (H)	3–4	4	7		Marl–limestone alternation	Bathyal zone	[161]
219				Hauterivian-Valanginian	Alexander island, Antarctica	S 70.8 °, E 68.5 °	-63.3						Sandstone and shale	Offshore	[154], PD

220	Mesozoic	Cretaceous	Early	Hauterivian	La Charce, France	N 44°28', E 5°27'	35.56	Planar spreite (S)	3–5	1		30×60×c	Marl–limestone alternation	Bathyal zone	[157-159,162]
221				Valanginian	Perneck, Austria	N 47.7°, E 13.7°	32.8	J-form spiraling spreiten (H)				6×8×c	Sandstone	Bathyal zone	[163], PD
222				Valanginian	Section KB1-A, Austria	N 47.9°, E 14.4°	32.6						Grey wackestone	Bathyal zone	[164], PD
223				Valanginian	Section KB1-A, Austria	N 47.9°, E 14.4°	32.6						Grey, argillaceous limestone	Bathyal zone	[164], PD
224				Valanginian	Bed 3, Section KB1-A, Austria	N 47.9°, E 14.4°	32.6						Grey, argillaceous limestone	Bathyal zone	[164], PD
225				Valanginian	Bed 9, Section KB1-A, Austria	N 47.9°, E 14.4°	32.6						Grey, argillaceous limestone	Bathyal zone	[164], PD
226				Valanginian	Bed 11, Section KB1-A, Austria	N 47.9°, E 14.4°	32.6						Grey, argillaceous limestone	Bathyal zone	[164], PD
227				Valanginian	Bed 13, Section KB1-A, Austria	N 47.9°, E 14.4°	32.6						Grey, argillaceous limestone	Bathyal zone	[164], PD
228				Valanginian	Bed 15, Section KB1-A, Austria	N 47.9°, E 14.4°	32.6						Grey, argillaceous limestone	Bathyal zone	[164], PD
229				Valanginian	Bed 17, Section KB1-A, Austria	N 47.9°, E 14.4°	32.6						Grey, argillaceous limestone	Bathyal zone	[164], PD
230				Valanginian	Bed 19, Section KB1-A, Austria	N 47.9°, E 14.4°	32.6						Grey, argillaceous limestone	Bathyal zone	[164], PD
231				Valanginian	Bed 25, Section KB1-A, Austria	N 47.9°, E 14.4°	32.6						Gray, argillaceous limestone	Bathyal zone	[164], PD
232				Valanginian	Bed 27, Section KB1-A, Austria	N 47.9°, E 14.4°	32.6						Grey, argillaceous limestone	Bathyal zone	[164], PD
233				Valanginian	Bed 29, Section KB1-A, Austria	N 47.9°, E 14.4°	32.6						Grey, argillaceous limestone	Bathyal zone	[164], PD
234				Valanginian	Bed 31, Section KB1-A, Austria	N 47.9°, E 14.4°	32.6						Grey, argillaceous limestone	Bathyal zone	[164], PD
235				Valanginian	Dobresti, Southern Carpathians, Romania	N 45°08'47.33", E 25°07'28.66"	35.77	U-form spiraling spreiten (H) and planar spreite (S)	5		2	10×8×c	Limestone	Bathyal zone	[165,166]
236				Valanginian	Butkov quarry, Ladce, Slovakia	N 49°2.67', E 18°31.69’	31.31	Planar spreite (S)	4				Bioturbated limestone	Bathyal zone	[167]

237	Mesozoic	Cretaceous	Early	Valanginian	Polomec quarry, Lietavská Lúčka, Slovakia	N 49°72', E 18°71'	41.396	Planar spreite (S)	4				Bioturbated limestone	Bathyal zone	167
238				Valanginian-Albian	Río Guanaco area, Agentina	S49°57.18', W 72°4.93'	-48.95						Black shales and marls	Bathyal zone	168
239				Berriasian	Well 2287, Pervomaiskaya area, Russia	N 59°27.53', E 75°05.8'	57.12	Planar spreite (S)	0.1–1				Mudstone	Bathyal zone	169
240				Berriasian	Tyumenskaya supperdeep well 6, Russia	N 66°15.7', E 77°37.5'	63.93	Planar spreite (S)	0.1–1				Clayey–siliceous black shale	Bathyal zone	169
241				Berriasian	Well 2, Boltnaya area, Russia	N 58°37.3', E 76°29.9'	56.52						Clayey–siliceous black shale	Bathyal zone	169
242				Berriasian	Castillon, France	N 43°53', E 6°30'	34.95	J-form spiraling spreiten (H)	3–5	8	3	30×60×c	Marl–limestone alternation	Bathyal zone	157-159
243				Berriasian	La Motte Chalacon, France	N 44°28', E 5°26'	35.56		3–5			30×40×c	Marl–limestone alternation	Bathyal zone	157-159
244				Berriasian?	Lioux, France	N 43°53', E 6°20'	35		3–5			30×40×c	Marl–limestone alternation	Bathyal zone	157-159
245				Berriasian?	Taloire, France	N 43°49', E 6°26'	34.93		3–5			30×40×c	Marl–limestone alternation	Bathyal zone	157-159
246		Jurassic	Late	Tithonian	Gucuo, Nielamu,Tibet, China	N 28.7827°, E 86.3408°	-43.6	U-form spiraling spreiten (H)	2–4			20×30×c	Calcareous siltstone or micrite	Lower offshore	170
247				Kimmeridgian	Wadi Laban, Saudi Arabia	N 23°50', E 45°08'	-1.87	U-form spiraling spreiten (3)	3–4	4	4	5×8×c	Limestone	Shelf	171
248				Oxfordian	Kamaguna section,Jhura Dome, North of Kachchh, India	N23°22'41", E 69°33'73"	-23.85	U-form spiraling lobate spreiten (H)	2–4	2.5		10×24×c	Limestone and marl	Upper offshore	172
249				Oxfordian	Pellecchia mounts, Italy	N 42°04', E 12°43'	30.11	J-form spiraling spreiten (H)	2–3			30×40×c	Marly limestone	Lower offshore	71,173
250				Callovian-Kimmeridgian	Qinghai, China	N 33.1°, E 91.8°	23.6						Siliciclastics	Coastal plain	174, PD
251			Middle	Bathonian	Sea cliffs near santa mera, Spain	N 43°31', W 5°20'	43.51	Helicoidal conical spreiten (H)	2–3		3		Marly limestone	Open shelf	97
252				Bathonian	South-Eastern France	N 43°53', E 6°20'	35	J-form spiraling spreiten (H)	3–5	5	2	30×40×c	Marl–limestone alternation	Continental slope	175
253				Bathonian	Chasteuil, France	N 43°50', E 6°25'	34.78	J-form spiraling spreiten (H)	3–5	3	3	40×120×c	Marl–limestone alternation	Lower Shelf	157-159,176,177

254	Mesozoic	Jurassic	Middle	Bathonian?	Teillon, France	N 43 °50', E 6 °25'	34.78	J-form spiraling spreiten (H)	3–5	2	2	40×120×c	Limestone	Lower Shelf	157-159,176,177
255				Bathonian	Ravin du Bès section, Bas Auran area, Subalpine Basin, SE France	N 43°57.63', E 6°18.92'	34.92						Limestone–marl alternation	Bathyal zone	178
256				Bathonian	Ravin d'Auran Section,Bas Auran area, Subalpine Basin, SE France	N 43°57.35', E 6°18.93'	34.91						Limestone–marl alternation	Bathyal zone	178
257				Bajocian	Ravin du Bès section, France	N 43°56.80', E 6°17.92'	34.90						Limestone	Bathyal zone	179
258				Bajocian	Piening Klippen belt, inner and outer Carpathians, Poland	N 50 °14', E 20 °01'	42.1	Planar spreite (S)	2–4	0.4	2	12×20×c	Limestone	Bathyal zone	180
259				Bajocian?	Cadières de Brandis, France	N 43 °50', E 6 °26'	34.78		3–5			40×120×c	Limestone	Lower Shelf	157-159,176,177
260				Bajocian?	Chabrières, France	N 44 °00', E 6 °41'	34.97		3–5			40×52×c	Limestone	Lower Shelf	157-159,176,177
261				Bajocian?	Pierlas, France	N 44 °01', E 7 °02'	34.99		3–5	3	2	40×53×c	Limestone	Lower Shelf	157-159,176,177
262				Bajocian-Bathonian	de la Praia da mareta, Algarve, Portugal	N 39 °29', W 8 ° 05'	33.23	J-form spiraling spreiten (H)	2–4	4	3		Limestone	Lower offshore	181
263				Bajocian-Bathonian	Jumara Dome, Kachchh Basin, India	N 22°38'10", E 70°50'22"	-21.78	J-form spiraling spreiten (H)	2–4		2	17×30×c	Sandstone	Ramp (upper offshore)	182
264				Bajocian-Bathonian	Jara Dome, Kachchh Basin, India	N 22°41'20", E 70°46'33"	-21.71	J-form spiraling spreiten (H)	3–5		2	10×26×c	Sandstone	Ramp (upper offshore)	182
265				Bajocian	Delisa, Vienna, Austria	N 48.216°, E 16.374°	32.94	Spirally coiled sprieten (H)					Limestone	Lower offshore	183
266				Bajocian	La Baume, Castellane, France	N 43°53'28", E 6°30'02"	32.77						Limestone–mal alternations	Platform slope	184
267				Bajocian	Khechem el kelb section, Tunisia	N 35°08'49.75", E 9°41'44.29"	24.17						Limestone–mal alternations	Bathyal zone	185
268				Bajocian	Chaabet el Attaris, Turnisia	N 35°14'21.35", E 9°42'21.09"	24.25						Limestone–mal alternations	Bathyal zone	185
269				Bajocian	Kef el Hassine, Turnisia	N 35°16'05.36", E 9°41'44.61"	24.28						Limestone–mal alternations	Bathyal zone	185

270	Mesozoic	Jurassic	Middle	Bajocian	C1, the Middle Atlas, Couches du Selloum, Morocco	N 33 °02'31.48", W 5 °21'22.69"	28.14						Limestone–mal alternations	Upper offshore	186
271				Bajocian	C2, the Middle Atlas, Couches du Selloum, Morocco	N 33 °03'45.88", W 5 °21'03.59"	28.146						Limestone–mal alternations	Upper offshore	186
272				Bajocian	C3, the Middle Atlas, Couches du Selloum, Morocco	N 33 °04'12.07", W 5 °20'37.09"	28.149						Limestone–mal alternations	Upper offshore	186
273				Bajocian	C4, the Middle Atlas, Couches du Selloum, Morocco	N 33 °04'52.09", W 5 °19'58.15"	28.155						Limestone–mal alternations	Upper offshore	186
274				Bajocian	C6, the Middle Atlas, Couches du Selloum, Morocco	N 33 °06'35.14", W 5 °16'15.59"	28.154						Limestone–mal alternations	Upper offshore	186
275				Bajocian	C7, the Middle Atlas, Couches du Selloum, Morocco	N 33 °07'22.90", W 5 °15'27.40"	28.16						Limestone–mal alternations	Upper offshore	186
276				Bajocian	C9, the Middle Atlas, Couches du Selloum, Morocco	N 33 °08'02.15", W 5 °14'20.31"	28.16						Limestone–mal alternations	Upper offshore	186
277				Bajocian	C11, the Middle Atlas, Couches du Selloum, Morocco	N 33 °04'44.34", W 5 °20'23.71"	28.155						Limestone–mal alternations	Upper offshore	186
278				Aalenian-Bajocian	Blumens tein, Swiss	N 46 °43', E 7 °30'	37.82	U-form spiraling spreiten (H)	2–3				Limestone	Upper offshore	187
279				Aalenian?	Soleilhas, France	N 43 °51', E 6 °38'	34.8		3–5			40×50×c	Limestone	Carbonate platform (shelf)	157-159,176
280				Aalenian-Bajocian	Chaudon, digne, France	N 44 °02', E 6 °18'	34.98		3–5	4	3	a×40×c	Limestone	Carbonate platform (shelf)	157-159,176
281				Toarcian-Aalenian	Porto de Mos, Portugal	N 39 °36'02.10", W 8 °48'54.10"	30.81						Marl-limestone alterations	Bathyal zone	188
282				Toarcian-Aalenian	Alvaiazere, Portugal	N 39 °49'28.07", W 8 °22'52.12"	30.84						Marl-limestone alterations	Bathyal zone	188
283				Toarcian-Aalenian	Rabacal, Portugal	N 40 °01'59.33", W 8 °27'45.94"	31.06						Marl-limestone alterations	Bathyal zone	188
284				Toarcian-Aalenian	Cantanhede, Portugal	N 40 °20'40.76", W 8 °35'35.62"	31.38						Marl-limestone alterations	Bathyal zone	188

285	Mesozoic	Jurassic	Middle	Aalenian	Western border of Iberia, lusitanian basin, Portugal	N 39 °22', W 9 °22'	30.76						Marl-limestone alterations	Upper offshore?	[188]
286			Early	Toarcian	Valdorbia section, Perugia, Italy	N 43 °26', E 12 °41'	30.06	Planar spreite (S)	2–4	3–4	3	10×35×c	Marly limestone	Lower offshore	[189]
287				Sinemurian-Pliensbachian	Kriza of the Tatra Mts, between Krakow and Vien, Slovakia	N 49 °08'04", E 19 °59'18"	29.04	Planar spreite (S)	2–5	2	3		Limestone	Bathyal zone	[190]
288				Sinemurian-Pliensbachian	Tatra Mts, Eastern ALPS, Swiss	N 46 °35', E 9 °27'	33.89	Planar spreite (S)	2–5	2	3		Marl and limestone	Bathyal zone	[190]
289				Hettagian	Trentino-Alto Adige, Italy	N 45.9 °, E 11.1 °	31.9						Mudstone and wackestone	Peritidal	[191], PD
290				Hettagian	Trentino-Alto Adige, Italy	N 45.9 °, E 11.0 °	31.9						Mudstone and wackestone	Peritidal	[192], PD
291				Hettagian	Trentino-Alto Adige, Italy	N 45.8 °, E 11.1 °	31.9						Wackestone	Peritidal	[193], PD
292				Hettagian	Lavini di Marco, Rovereto, Italy	N 45 °49', E 11 °03'	32.57	J-form spiraling circular spreiten (H)	2–5	1	3	8.6×14.4×c	Wackestone and mudstone	Tidal-flat	[194]
293		Triassic	Late	Rhaetian	Roaring Bay, South Otago, New Zealand	S 46 °26', E169 °48'	-74.41	J-form spiraling spreiten (H)	3–4	2	2	8×15×c	Sandstone	Shelf	[195]
294				Rhaetian	J. Wahrah, Oman	N 23 °13.24', E 56°54.44’	-3.07	Rooster-tail-like spreiten (H)				15×10×c	Sandstone	Bathyal zone	[196]
295				Norian	Mt charon on the Hanmer Range,North Canterbury, New Zealand	S 42 °29', E 172 °45'	-74.91	J-form spiraling spreiten (H)	2–3	0.7	2		Lime mudstone	Lower offshore	[197]
296				Carnian?	Qumalai-Xieduo, Qinghai, China	N 34.10 °, E 95.45 °	29.04	J-form spreiten (incomplete) (H)	1–3			8×14×c	Siltstone or fine sandstone	Bathyal zone	[198]
297				Carnian	Ga'a'ma, Nandamu region, Rangtang, Sichuan, China	N 32.3906 °, E 101.0934 °	36.22						Sandstone	Bathyal zone	[199]
298				Carnian	Maladun, Songpan, Sichuan, China	N 33.06 °, E 102.92 °	37						Siltstone	Bathyal zone	[200]
299			Middle	Ladinian	Monte San Giorgio, Switzerland	N 45 °54.51', E 8°57.97’	7.95						Limestone	Bathyal zone	[201]
300				Anisian-Ladinian, maybe Ladinian	Hotzdorf, Germany	N 50 °57', E 11 °17'	42.19	U-form spreiten (H)	4	6	2	28×34×c	Calcilutites	Carbonate ramp	[202]
301				Anisian-Ladinian, maybe Ladinian	Kara-Korum ranges, Xingjiang, China	N 35 °24'53", E 78 °58'59"	37.15	J-form spiraling circular spreiten (H)	2–3			10×35×c	Siltstone	Bathyal zone	[203]

302		Triassic	**Middle**	Anisian-Ladinian, maybe Ladinian	Gelmeroda, Germany	N 50 °56'42", E 11 °17'20"	17.44	Lobe like spreiten (H)	3–4	0.5	1	25×10×c	Micritic limestone	Carbonate ramp	[204]
303			**Early**	Olenekian?	Middle Buntsandstein, Ruhr region, Germany	N49 °35', E9 °01'	15.65	U-form spiraling spreiten (H)	3–5		3	20×16×c	Red sandstone	Nearshore	[205]
304	Paleozoic	Permian	**Lopingian**	Changhsingian	Dongpan, Guangxi, China	N 23 °33', E 107 °40'	-2.45	Planar spreite (S)	2–3	0.3	1	3×5×c	Siliceous rock	Continental slope	OC
305				Changhsingian	Shangsi, Sichuna, China	N 32 °20.158', E 105 °26.546'	2.71	Planar spreite (S)					Limestone	Lower offshore-slope	[206]
306				Changhsingian	Lilang, lingti, Gungri, Kidul, Muth and Po sections, India	N 32 °24', E 78 °32'	-20.96	Planar spreite (S)	3–5			a×50×c	Black shale	Shelf	[207]
307				Changhsingian	Persian Gulf, Iran	N 26 °15', E 52°14’	-28.75	Planar spreite (S)	4				Mudstone	Subtidal zone	[208,209]
308				Changhsingian	Salbard, Spitsbergen, Norway	N 78 °2.23', E 13 °42.83'	41.15						Cherty shale	Abyssal zone	[210]
309				Changhsingian	Dolomites region, Northern Italy	N 46 °15.13', E 11 °31.99'	3.28						Limestone	Subtidal zone	[211]
310				Wuchiapingian?	Brooks Range, Alaska, USA	N 69 °16', W 144°01’	47.19	Helicoildal spreiten (H)				30×20×c	Sandy siltstone	Inner shelf	[212]
311			**Guadalupian**	Capitantian-Wuchiapingian	Sverdrup Basin, Hvitland Peninsula, Canada	N 81 °05', W 88 °33'	41.14						black siliceous, siltstone, shale	mid- to outer-ramp	[213]
312				Capitanian	Iran	N 32.9 °, E 50.1 °	-22.8						Limestone	Shallow subtidal	[214], PD
313				Capitanian	Svalis Dome, Barents Sea, Norway	N 73 °20.55', E 23 °14.42'	37.98						Cherty mudstone	Abyssal zone	[215]
314				Capitanian	Black head, sydney basin, Australia	S 32 °04', W 152 °32'	-59.28	Planar spreite (S)	3–9	0.9	2		Greywacke	Nearshore	[216,217]
315				Wordian	Huaf-Haushi Uplift of Interior, Oman	N 21 °00', E 57 °40'	-34.89	J-form spiraling spreiten (H)	2–4	0.4	2		Limestone	Nearshore	[218]
316				Roadian	Laibin, China	N 23 °43', E 109 °13'	-1.36	Planar spreite (S)	2–4	0.4	2		Wackstone	Slope	[216,219-221]
317				Roadian	Heshan Matian, Guangxi, China	N 23 °48', E 108 °52'	-1.52	Planar spreite (S)	1–4	0.4	2		Limestone	Lower offshore	[222]
318				Roadian	Shangsi, Sichuna, China	N 32 °20.158', E 105 °26.545'	2.71	U-form spiraling spreiten (H)	4	4		4×9×c	Limestone	Upper offshore	[206]

319	**Paleozoic**	**Permian**	**Guadalupian**		Roadian	Sverdrup basin, Canada	N 79 °30',<br>W 80 °02'	27.97	J-form spiraling spreiten (H)	3–5		2		Sandstone	Shoreface	223
320					Roadian	Laibin, China	N 23 °43',<br>E 109 °13'	-8.59	Planar spreite (S)	2–4	4	3		Greywacke	Lower offshore	219
321			**Cisuralian**		Kungurian	Qixingjie, Lianyuan, Hunan, China	N 27.9015 °,<br>E111.8581 °	-4.84	Planar spreite (S)	2–4				Wackestone	Lower offshore	224
322					Artinskian	Ishabel Formation, western Alberta, Canada	N53 °54',<br>W17 °21'	53.9	Swirled, low conical mounds of radiationg filaments					Silty andstone and sandy limestone	Offshore	225,226
323					Artinskian	Central Alborz, Iran	N 35 °48.5',<br>E 51°8.6’	-19.68	J-form spiraling srpeiten (H)				10×12×c	Limestone with chert	Open marine（Shelf）	227
324					Asselian	Satpura, Gondwana basin, India	N 22 °28',<br>E 78 °14'	-54.14	Planar spreite (S)	3–4	0.4	1	13×27×c	Fine sandstone and mudstone	Lower shoreface to inner shelf	228
325					Asselian	Xizhangzhuang, Jiaozuo, China	N 35 °14',<br>E 112 °54'	17.62	Planar spreite (S)	2–3	3	3		Limestone	Shelf margin	229,230
326					Asselian	Ranifanj Basin, India	N 23 °45',<br>E 86 °42'	-70.31	Flat spiral spreiten (H)	2–9	5	4	8×15×c	Silty mudstone	Shelf	231
327		**Carboniferous**	**Pennsylvanian**	**Late**	Kasimovian	Utah, USA	N 40 °26'23.98",<br>W 112 °06'21.21"	7.1	Planar spreite (S)	2–3		1	8×12×c	Sandstone	Bathyal zone	232
328					Kasimovian	Kansas Edmonds Core No.1A, USA	N 39 °01'15.48",<br>W 98 °25'29.38"	-0.42	Planar spreite (S)	2–3				Grey shale	Offshore	233
329					Kasimovian-Gzhelian	Carnic Alps, Italy	N 46 °31',<br>E 13 °02'	-10.54	Helicoidal spreiten (H)	3–7		3	30×40×c	Sandstone	Lower offshore	234
330					Kasimovian	Oquirrh Basin, Utah, USA	N 41 °16',<br>W111 °59'	3.45	Helicoidal spreiten (H)	2–3		1	12×10×c	Sandstone	Bathyal zone	232
331				**Middle**	Moscovian	Priokskii, Voskresensk -Ozery-Kolomna, Russia	N 55 °03'47.44",<br>E 38 °51'11.91"	15.42						Mudstone, packstone–wackestone	Open subtidal	235
332					Moscovian-Kasimovian	Utah, USA	N 40 °10'10.73",<br>W 111 °32'34.47"	6.61	U-form spiraling spreiten (H)				8×13×c		Bathyal zone	232
333					Moscovian	Cape Breton, Nova Scotia, Canada	N 46 °19',<br>W 60 °17'	-8.93	J-form spiraling spreiten (H)	6–10		1	a×24.5×c	Sandstone	Tidal flat	236
334					Moscovian	Ciyao, Jingyuan, China	N 36.80 °,<br>E 104.95 °	22.16	J-form spiraling spreiten (H) and planar spreite (S)	2–3			10×10×c	Pelsparite	Middle nearshore	237

335	Paleozoic	Carboniferous	Pennsylvanian	Middle	Moscovian	Utah, USA	N 40 °12'19.79",<br>W 111 °30'26.14"	2.419	U-form spiraling spreiten (H)	2–3		2	8×14×c		Bathyal zone	232
336				Early	Bashkirian	Jungar Basin, China	N 44 °38',<br>E 89 °54'	40.55	Planar spreite (S)	2–3	0.3			Mudstone and sandstone	Bathyal zone	238
337					Bashkirian	Guadalupe Box, Gilman, USA	N 35 °43',<br>W 106 °45'	0.77	U-form spiraling spreiten (H)	2–3		2	21×30×c	Calcareous siltstone	Shelf	239
338					Bashkirian	Northeastern Kentucky, USA	N 38 °17',<br>W 83 °20'	-6.62						Sandstone	Nearshore	240
339					Bashkirian	Duku road, Jungar Basin, Xingjiang, China	N 43.77 °,<br>E 84.4 °	30.8						Marlite	Bathyal zone	241
340					Bashkirian	Northern Tennesse, USA	N 36 °27',<br>W 86 °05'	-12.45	U-form spiraling spreiten (H)	2–3	0.3	2	10×14×c	Sandstone	Upper offshore	242
341					Bashkirian	Within the Walker and Fayette Counties, Black warrior basin, Alabama, USA	N 33 °53',<br>W 87 °29'	-9.23						Sandstone	Upper offshore	243
342			Mississippian	Late	Serpukhovian	Caldas de san Adrian, Leon, Spain	N 43 °09',<br>W 5 °37'	-3.69	J-form sprialing spreiten (H)	2–3	0.3	2	3×6×c	Micritic limestone	Slope	97
343					Serpukhovian	Xikuangshan, Hunana, China	N 27.778 °,<br>E111.495 °	-10.47						Wackstone	Lower offshore	244
344					Serpukhovian	Shuizutang, Lianxian, Guangdong, China	N 24.622 °,<br>E 112.37941 °	-9.42	Planar spreite (S)	5				Marlite	Lower offshore	245
345					Serpukhovian	Malanbian Shuiku, Xinshao, Hunan, China	N 27 °21',<br>E 111 °25'	-12.58		1–3	0.3	1	7×20×c	Bioclastic limestone	Offshore	246
346					Serpukhovian	Malanbian Shuiku, Xinshao, Hunan, China	N 27 °21',<br>E 111 °25'	-13.02				1	8×15×c	Limestone	Tidal flat	246
347					Serpukhovian	Asturias, Spain	N 43 °14',<br>W 5 °58'	-14.61	Helicoidal spreiten (H)	2–3		1	8×14×c	Sandstone	Nearshore	97
348				Middle	Visean	Gilf Kebir-Abu Ras area, Egypt	N 23 °35',<br>E26 °23'	-38.29	U-form spiraling spreiten (H)	2–3		1	7×16×c	Silty shale	Upper offshore	247
349					Visean	Dushan Ercengpo, Guizhou, China	N 25 °47',<br>E 107 °17'	-14.05	Planar spreite (S)	5–6	0.5			Limestone	Upper offshore	OC
350					Visean	Luochen, Guangxi, China	N 24 °47',<br>E 108 °51'	-12.36		2–4		2	15×20×c	Dark limestone	Upper offshore	248

351	Paleozoic	Carboniferous	Mississippian	Middle	Visean	Huxian Chini, Guangdong, China	N 23°27', E 113°13'	-8.64		3–5	0.5	2	10×15×c	Silty shale	Littoral zone	[249]
352					Visean	Hejialong, Luguan, Xinhua, Hunan, China	N 27°45', E 111°11'	-14.73	J-form spiraling spreiten (H)			2	11×16×c	Limestone	Shoreface	[250]
353					Visean	Shuichangbaoxiang, Longli, Guizhou, China	N 26°25', E 106°56'	-14.72	Spiraling tongue-like spreiten (H)	2–3		2	10×15×c	Dark limestone	Lower offshore	[251]
354					Visean	Dayu, Guiling, Guangxi, China	N 25°13', E 110°21'	-11.75	Spiraling tongue–like spreiten (H)	2–3		2	12×20×c	Bioclastic limestone	Upper offshore	[252]
355					Visean	Heatherslade Bay, Horizon 11, Wales, UK	N 51.6°, W 4.0°	-10.7						Shelly, packstone and mudstone	Shallow subtidal	[214], PD
356					Visean	Heatherslade Bay, Horizon 13, Wales, UK	N 51.6°, W 4.0°	-10.7						Shelly, packstone and mudstone	Shallow subtidal	[214], PD
357					Visean	Heatherslade Bay, Horizon 17, Wales, UK	N 51.6°, W 4.0°	-10.7						Shelly, packstone and mudstone	Shallow subtidal	[214], PD
358					Visean	Ogmore by Sea, Horizon 31, Wales, UK	N 51.6°, W 3.0°	-10.7						Shelly, packstone and mudstone	Shallow subtidal	[214], PD
359					Visean	Ogmore by Sea, Horizon 32, Wales, UK	N 51.6°, W 3.0°	-10.7						Shelly, packstone and mudstone	Shallow subtidal	[214], PD
360					Visean	Ogmore by Sea, Horizon 34, Wales, UK	N 51.6°, W 3.0°	-10.7						Shelly, packstone and lime mudstone	Shallow subtidal	[214], PD
361					Visean	Ogmore by Sea, Horizon 35, Wales, UK	N 51.6°, W 3.0°	-10.7						Shelly, packstone and lime mudstone	Shallow subtidal	[214], PD
362					Visean	Ogmore by Sea, Horizon 37, Wales, UK	N 51.6°, W 3.0°	-10.7						Shelly, packstone and lime mudstone	Shallow subtidal	[214], PD
363					Visean	Ogmore by Sea, Horizon 42, Wales, UK	N 51.6°, W 3.0°	-10.7						Silty lime mudstone	Peritidal	[214], PD
364					Visean	Malý Rabštýn, Czech Republic	N 49°40', E 17°23'	-6.83	Irregular lobate spreiten (H)	1.5–2.5			a×15×c	Turbidite	Bathyal zone	[253]
365					Visean	Indiana, USA	N 39.5°, W 86.5°	-19						Sandstone and shale	Delta plain	PD
366					Visean	Xikuangshan, Hunana, China	N 27.778°, E111.495°	-12.85	Spiral spreiten (H)					Wackestone	Lower offshore	[244]
367					Visean	Hejialong, Luguan, Xinhua, Hunan, China	N 27°45', E 111°11'	-13.92						Limestone`	Upper offshore	[250]

368	Paleozoic	Carboniferous	Mississippian	Middle	Visean	Paprotnia, Poland	N 52°12.28', E 20°25.41'	-4.62		1–5	3.5		16×7×3.5	Mudstone	Lower offshore	254
369				Early	Tournaisian-Visean	Tournai, Belgium	N 50°30', E 3°16'	2.37	J-form spiraling spreiten (H)	2–3	5	3	45×90×c	Limestone	Carbonate ramp	255
370					Tournaisian	Zhongpaixiang, Longli, Guizhou, China	N 26°28', E 106°57'	-14.72	Spiral spreiten (H)	2–3		3	10×35×c	Siltstone	Shoreface	251
371					Tournaisian	Mingzhuxiang, Longli, Guizhou, China	N 26°28', E 106°59'	-14.72	Spiraling circular spreiten (H)	2–3		2	30×45×c	Siltstone	Littoral zone	251
372					Tournaisian	Yvoir, Tournai, Belgium	N 50°19', E 4°52'	-9.01	Spiral spreiten (H)	2–3		3	a×40×c	Carbonate	Carbonate ramp	256
373					Tournaisian	western Montana, Utah, USA	N 41°13', W 112°18'	-1.41	U-form spiraling spreiten (H)	3–8		3	a×28×c	Limestone	Offshore	257
374					Tournaisian	Ogmore by sea, Swansea, South Wales	N 51°28'31.29", W 3°42'22.39"	-9.021	Regular spiral spreiten (H)				a×15×c	Packstone	Lower offshore	258
375					Tournaisian	Three Cliffs Bay, South Wales	N 51°34'19.25", W 4°06'53.51"	-8.96	Arcute spiral spreiten (H)				a×8×c	Graded limestone	Lower offshore	258
376					Tournaisian	Indiana, USA	N 39.8°, W 86.7°	-20						Siltstone	Shelf	PD
377					Tournaisian	Shuizutang, Lianxian, Guangdong, China	N 24.622°, E 112.37941°	-1.4	Planar spreite (S)	2.6–4	0.4			Wackestone	Upper offshore	245
378					Tournaisian	Chile	S 24.5°, W 69.5°	-64.4						Sandstone and shale	Nearshore	PD
379		Devonian	Late		Famennian	Cuyahoga County, Ohio, USA	N 41.5°, W 81.7°	-30.7						Shale and siltstone	Offshore	PD
380					Famennian	Cuyahoga County, Ohio, USA	N 41.6°, W 81.6°	-30.6						Shale and siltstone	Offshore	PD
381					Famennian	Kufra Basin, Libya	N 22°23', E24°07'	-51.23	J-form irregular spiraling spreiten (H)	3		1	a×40×c	Sandstone	Tidal	259
382					Famennian	Zhongcun, Shanyang, Shanxi, China	N 33.469°, E 110.212°	-10.23						Siltstone	Bathyal zone	260
383					Frasnian-Famennian	Guodingshan, Hanyang, China	N 30°34', E 114°10'	-2.06	U-form spiraling spreiten (H)	2–3		1	7×15×c	Sandstone	Lower shoreface	OC
384					Frasnian-Famennian	New York, USA	N 40°38', W 73°58'	-37.74	U-form / arcute spiraling spreiten (H)	2–3		1	10×18×c	Sandstone	Upper offshore	242,261

385	**Paleozoic**	**Devonian**	**Late**	Frasnian	Zhongcun, Shanyang, Shanxi, China	N 33.469°, E 110.212°	-9.19						Siltstone or calcareous sandstone	Bathyal zone	260
386				Frasnian	Hougaoping, Guangyuan, Sichuan, China	N 32°20', E 105°26'	-7.03	Spiraling circular spreiten (H)	1–2	0.2	1	3×7×c	Muddy siltstone	Upper offshore	262
387				Frasnian	Aragon, Spain	N 41.0°, W 1.0°	-34.8						Shale and sandstone	Shelf	PD
388				Frasnian	Northern Illinois, Gas #1 MAK Core, 1141.ft, USA	N 41°04'42.12", W 90°16'26.04"	-40.116	Planar spreite (S)	2–3			2×6×c	Bioturbated mudstone	Upper shelf	263
389			**Middle**	Givetian	New York, USA	N 42.8°, W 76.7°	-35.2					20×15×c	Calcareous sandstone	Lower nearshore	264
390				Givetian	North Cherry-Valley village, New YorK, USA	N 42.82°, W 74.78°	035.47	U-form spiraling coiled spreiten (H)				12×20×c	Calcareous sandstone	Lower nearshore	264
391				Givetian	Cayuga County, New York, USA	N 42.9°, W 76.5°	-37.2						Shelly, grey lime mudstone	Shallow subtidal	PD
392				Givetian	Onodaga County, New York, USA	N 42.9°, W 76.0°	-37.2						Shelly, grey wackestone	Shallow subtidal	PD
393				Givetian	Madison County, New York, USA	N 42.9°, W 75.9°	-37.3						Shelly, grey limestone	shallow subtidal	PD
394				Givetian	Onodaga County, New York, USA	N 42.9°, W 76.2°	-37.3						Shelly, grey, calcareous shale	lower shoreface	PD
395				Givetian	Cayuga County, New York, USA	N 42.8°, W 76.7°	-37.3						Grey lime mudstone	shallow subtidal	PD
396				Givetian	Onodaga County, New York, USA	N 42.9°, W 76.0°	-37.2						Shelly, grey limestone	Deep subbtidal shelf	PD
397				Givetian	Madison County, New York, USA	N 42.9°, W 75.9°	-37.3						Shelly, grey limestone	Deep subbtidal shelf	PD
398				Givetian	Cayuga County, New York, USA	N 42.9°, W 76.5°	-37.2						Shelly, grey, calcareous shale	Lower shoreface	PD
399				Givetian	Onodaga County, New York, USA	N 42.9°, W 76.4°	-37.2						Shelly, grey, calcareous shale	Lower shoreface	PD
400				Givetian	Madison County, New York, USA	N 42.8°, W 75.2°	-37.5						Grey, calcareous sandstone	Shoreface	PD
401				Givetian	Genesse County, New York, USA	N 43.0°, W 78.1°	-36.9						Grey, silty shale	Deep subtidal ramp	PD

402	Paleozoic	Devonian	Middle	Givetian	Livingston County, New York, USA	N 42.8 °, W 77.8 °	-37						Grey, silty shale	Deep subtidal ramp	PD
403				Givetian	Ontario Ounty, New York, USA	N 42.8 °, W 77.0 °	-37.2						Grey, calcareous shale	Deep subtidal ramp	PD
404				Givetian	Erie County, New York, USA	N 42.7 °, W 78.9 °	-37						Grey mudstone	Deep subtidal shelf	PD
405				Givetian	Livingston County, New York, USA	N 42.8 °, W 77.9 °	-37						Grey mudstone	Deep subtidal shelf	PD
406				Givetian	Dahekou, Dushan, Guizhou, USA	N 25 °50', E 107 °31'	-1.18	Helicoidal arcute spreiten (H)	4–12		1	17×20×c	Siltstone	Littoral zone	265
407				Givetian	Catskill Mts, east-central New York, USA	N 41 °59'50.45", W 74 °19'53.36"	-43.19	Helicoidal lobate spreiten (H)		0.4	1	a×20×c	Fine sandstone and siltstone	Nearshore to offshore	266
408				Givetian	Witteberg and bokkeveld series, ladismith district, South Africa	S 31 °19', E19 °04'	-62.23	Flat spirally coiled spreiten (H)	2–3	0.3	1	15×20×c	Sandstone	Littoral zone	267
409				Givetian	Witteberg and bokkeveld series, ladismith district, South Africa	S 31 °19', E19 °04'	-62.23	J-form spiraling spreiten (H)	2–3	0.3	1	18×20×c	Silty sandstone	Littoral zone	267
410				Givetian	Witteberg and bokkeveld series, ladismith district, South Africa	S 31 °19', E19 °04'	-62.23	J-form spiraling spreiten (H)	2–3	0.3	1	7×9×c	Sandstone	Littoral zone	267
411				Givetian	Witteberg and bokkeveld series, ladismith district, South Africa	S 31 °19', E19 °04'	-62.23	Flat spirally coiled sprieten (H)	2–3	0.3	1	14×21×c	Sandstone	Littoral zone	267
412				Givetian	North of Tamworth, NSW, Australia	S 31 °03', E 150 °55'	-12.83	Planar spreite (H)	2–3				Siltstone	Restricted neritic of moderate depth	268
413				Givetian	Palencia, Spain	N 43.0 °, W 0.9 °	-39						Limestone and shale	Offshore	PD
414				Givetian	Parnaiba Basin, Brazil	S7 °56.4', W 41°58.2’	-71.84	J-form spiraling spreiten (H)				11×10.5×c	Fine sandstone	Lower offshore	269
415				Eifelian	Wujia, Guanyang, Guangxi, China	N 25 °29', E 111 °05'	-1.73	J-form spiraling spireten (H)	1–3			15×20×c	Muddy siltstone	Shoreface	270
416				Eifelian	Tongtianshuiku, Linwu, Hunan, China	N 25 °18', E 112 °29'	-1.92	J-form spiraling spireten (H)	1–5		1	6×7×c	Siltstone	Shoreface	271,272
417				Eifelian	Luguan, Xinhua,Hunan, China	N 27 °45', E 111 °12'	-3.93	J-form spiraling spireten (H)			1	7×14×c	Siltstone	Shoreface	271,272

418	Paleozoic	Devonian	Middle	Eifelian	Banshan, ningyuan, China	N 25°24', E 111°52'	-1.15	Spirally coiled sprieten (H)	2–3	0.3	1	8×12×c	Siltstone	Lower shoreface	OC
419			Early	Emsian to Eifelian	Palencia, Spain	N 43.0°, W 0.9°	-44.1						Limestone–marl alternation	Offshore	PD
420				Emsian	Guangxi, China	N 22.0°, E 108.0°	3.8						Yellow mudstone	Bathyal zone	PD
421				Emsian	Luofo, Guangxi, China	N 22.0°, E 108.0°	3.8						Black, gray green mudstone	Bathyal zone	PD
422				Emsian	Palencia, Spain	N 42.9°, W 4.5°	-44.2						Shale and sandstone	Subtidal	PD
423				Emsian	Bohemia point, Czechoslovak	N 50°06', E 15°45'	-34.11	Helicoidal spreiten (H)	2–3	0.6			Bioclastic limestone	Lower offshore	[273]
424				Emsian	Ganxi, Beichuan, Sichuan, China	N 31°54', E 104°41'	-3.9	Spiraling circular/elliptical spreiten (H)	1–2	0.2	1	10×25×c	Siltstone	Upper offshore	OC
425				Emsian	Ganxi, Beichuan, Sichuan, China	N 31°51', E 103°40'	-3.56	U-form spiraling spreiten (H)	2–3	0.3	1	12×14×c	Muddy siltstone	Lower shoreface	OC
426				Emsian	Ganxi, Beichuan, Sichuan, China	N 31°51', E 103°40'	-3.56	J-form spiraling spreiten (H)	2–3	0.3	1	6×9×c	Muddy siltstone	Lower shoreface	OC
427				Emsian	Mountainville, Quaary Hill, New York, USA	N 41°24', W74°12'	-43.79	Flat spirally coiled spreiten (H)	3–4		1	15×30×c	Sandstone	Offshore-onshore cycle	[274]
428				Pragian	Long mountain, Eureka country, Nevada, USA	N 39°46', W 116°50'	-20.53						Limestone	Lower offshore	[275]
429				Pragian	Parana Basin, Brazil	S 24°48', W 51°24'	-76.24						Siltstone	Offshore	[276]
430				Pragian	Ponta crossa, Brazil	S 25°08', W 50°14'	-76.85						Siltstone and mudstone	Upper offshore	[277]
431				Pragian to Emsian	Palencia, Spain	N 43.0°, W 0.9°	-46.1						gray shale and micaceous, calcareous siltstone	Offshore	PD
432				Pragian	northwestern Texas, USA	N 31°34', W 102°27'	-41.28	Planar spreite (S)	5–6				Limestone	Offshore	[278]
433				Pragian	Anddes, Bolivia	S 17°12', W 67°50'	-64.05	Flat spirally coiled spreiten (H)	4–10		1	15×85×c	Sandstone	Offshore	[279]
434				Lochkovian	Liujing, Hengxian, Guangxi, China	N 22°53', E 108°52'	2.55	J-form spiraling spreiten (H)	1–2		1	7×10×c	Siltstone	Shoreface	[280]

435	**Paleozoic**			Lochkovian	Woodmont section, Maryland, USA	N 38°58', W 77°05'	-52.87						Chert	Upper offshore	20
436		**Silurian**	**Ludlow**	Ludfordian	Sierra del gallo, province salta, Agentina	S23°09', W 66°16'	-54.33	J-form spiraling spreiten (H)	3–4		1	8×15×c	Sandstone and siltstone	Lower offshore	281
437				Gorstian-Ludfordian	Quebec, Canada	N 48.2°, W 65.0°	-28.4						Argillaceous sandstone	Lower shoreface	PD
438			**Llandovery**	Aeronian	Sierra de Zapla, NW Agentina	S 38°51.18', W 69°48.63’	-42.84	J-form spiraling spreiten (H)				20×30×C	Siltstone	Muddy shelf (Lower offshore)	282,283
439		**Ordovician**	**Late**	Hirnantian	Arrow Canyon Range, South Nevada, USA	N 36°30', W 114°51'	-17.25	J-form spiraling spreiten (H)	1–3		1	5×16×c	Quartzite	Upper offshore	284
440				Sandbian	Seal Creek, Australia	S 37°39', E 149°41'	14.44	Planar spreite (S)	5–7				Chert	Bathyal zone	285
441			**Middle**	Darriwilian	Grondines, Quebec, Canada	N 46°14', W 72°01'	-29.793	U- or J-form spiraling spreiten (H)	3–4		1	5×10×c	Limestone	Shallow shelf	286
442				Dapingian	St. Lawrence Lowland, Canada	N 45°30', W 73°04'	-27.04	Flat spirally coiled spreiten (H)	3–5		1	6×8×c	Micrite and argillaceous calcisiltites	Shallow shelf	286
443				Dapingian	Wuhai, NW of Ordos Basin, China	N 39.3795°, E 106.8932°	-21.08	J-form spiraling spreiten (H)	3–5		1	8×14×c	Marlite	Bathyal zone	287
444		**Cambrian**	**Epoch 3**	Drumian?	Palác Hill, Czech	N 49°56', E 15°40'	-43.64	Planar spreite (S)	2–3				Siltstone	Lower offshore	288
445			**Epoch 2**	Age 3	Xuzhou, Jiangxu, China	N 34°03', E 117°03'	-4.89	Arcute spirally coiled spreiten (H)	4–5		1	13×10×c	Sandstone	Lower offshore	289
446				Age 3	Xuzhou, Jiangxu, China	N 34°03', E 117°03'	-4.89	Tongue-like spreiten (H)	3		2	15×b×c	Sandstone	Lower offshroe	289
447				Age 3	Tayshir I, Mongolia	N 46°40', E 96°29'	3.83	Circular spiraling spreiten					Sandstone	Lower shoreface	290
448			**Terreneuvian**	Fortunian	Death Valley region, California, USA	N 36°08.5', W 116°09'	5.13	Arcute spirally coiled spreiten (H)		0.6	1	8.1×b×c	Siltstone	Subtidal	291

Author contributions: Li-jun Zhang conceived the study. L. J. Zhang and R. Y. Fan contributed to the Phanerozoic *Zoophycos* database. All authors participated in data preparation, discussion and interpretation.

References and Notes

1. Ekdale, A. & Berger, W. Deep-sea ichnofacies: modern organism traces on and in pelagic carbonates of the western equatorial Pacific. *Palaeogeogr. Palaeoclimatol. Palaeoecol.* **23**, 263–278 (1978).
2. Wetzel, A. Recent bioturbation in the deep South China Sea: A uniformitarian ichnologic approach. *Palaios* **23**, 601–614 (2008).
3. Rodríguez-Tovar, F. J., Löwemark, L. & Pardo-Igúzquiza, E. *Zoophycos* cyclicity during the last 425ka in the northeastern South China Sea: Evidence for monsoon fluctuation at the Milankovitch scale. *Palaeogeogr. Palaeoclimatol. Palaeoecol.* **305**, 256–263 (2011).

4. Löwemark, L. Ethological analysis of the trace fossil *Zoophycos*: hints from the Arctic Ocean. *Lethaia* **45**, 290–298 (2012).
5. Löwemark, L., O'Regan, M., Hanebuth, T. J. J. & Jakobsson, M. Late Quaternary spatial and temporal variability in Arctic deep-sea bioturbation and its relation to Mn cycles. *Palaeogeogr. Palaeoclimatol. Palaeoecol.* **365**, 192–208 (2012).
6. Löwemark, L. & Werner, F. Dating errors in high-resolution stratigraphy: a detailed X-ray radiograph and AMS-^{14}C study of *Zoophycos* burrows. *Mar. Geol.* **177**, 191–198 (2001).
7. Löwemark, L. & Schäfer, P. Ethological implications from a detailed X-ray radiograph and ^{14}C study of the modern deep-sea *Zoophycos*. *Palaeogeogr. Palaeoclimatol. Palaeoecol.* **192**, 101–121 (2003).
8. Löwemark, L., Schönfeld, J., Werner, F. & Schäfer, P. Trace fossils as a paleoceanographic tool: evidence from Late Quaternary sediments of the southwestern Iberian margin. *Mar. Geol.* **204**, 27–41 (2004).
9. Löwemark, L. & Grootes, P. M. Large age differences between planktic foraminifers caused by abundance variations and *Zoophycos* bioturbation. *Paleoceanography* **19**, 1–9 (2004).
10. Löwemark, L., Schönfeld, J. & Schäfer, P. Deformation of pyritized burrows: A novel technique for the detection and estimation of core shortening in gravity cores. *Mar. Geol.* **233**, 37–48 (2006).
11. Leuschner, D. C., Sirocko, F., Grootes, P. M. & Erlenkeuser, H. Possible influence of *Zoophycos* bioturbation on radiocarbon dating and environmental interpretation. *Mar. Micropaleontol.* **46**, 111–126 (2002).
12. Löwemark, L. *et al.* Ethology of the *Zoophycos*-Producer: Arguments Against the Gardening Model from $\delta^{13}C_{org}$ Evidences of the Spreiten Material. *T. A. O. S.* **15**, 713–725 (2004).
13. Löwemark, L., Lin, H. L. & Sarnthein, M. Temporal variations of the trace fossil *Zoophycos* in a 425 ka long sediment record from the South China Sea: Implications for the ethology of the *Zoophycos* producer. *Geol. Mag.* **143**, 105–114 (2006).
14. Löwemark, L., Lin, I. T., Wang, C. H. & Schönfeld, J. A test of the gardening hypothesis for the trace fossil *Zoophycos*. *Soc. Sediment. Geol.* **88**, 79–86 (2007).
15. Wetzel, A. Deep-sea ichnology: Observations in modern sediments to interpret fossil counterparts. *Acta Geol. Pol.* **60**, 125–138 (2010).
16. Mosher, D. C., Moran, K. & Hiscott, R. N. Late Quaternary sediment, sediment mass flow processes and slope stability on the Scotian Slope, Canada. *Sedimentology* **41**, 1039–1061 (1994).
17. Wetzel, A., Tjallingii, R. & Wiesner, M. G. Bioturbational structures record environmental changes in the upwelling area off Vietnam (South China Sea) for the last 150,000 years. *Palaeogeogr. Palaeoclimatol. Palaeoecol.* **311**, 256–267 (2011).
18. Beiersdorf, H. & Natland, J. H. Sedimentary and diagenetic processes in the gentral panama basin since the late miocene: the lithology and composition of sediments from deep sea drilling project sites 504 and 505. *DSDP* **69**, 343–383 (1979).
19. Chamberlain, C. K. Trace fossils in DSDP cores of the Pacific. *J. Paleontol.* **49**, 1074–1096 (1975).
20. Seilacher, A. Bathymetry of trace fossils. *Mar. Geol.* **5**, 413–428 (1967).
21. Wetzel, A. & Werner, F. Morphology and ecological significance of *Zoophycos* in deep-sea sediments off NW Africa. *Palaeogeogr. Palaeoclimatol. Palaeoecol.* **32**, 185–212 (1981).
22. Wetzel, A. Ecologic interpretation of deep-sea trace fossil communities. *Palaeogeogr. Palaeoclimatol. Palaeoecol.* **85**, 47–69 (1991).
23. Fu, S. & Werner, F. Is *Zoophycos* a feeding trace. *Neues Jahrb. Geol. Palaontol. Abh.* **195**, 37–47 (1995).
24. Fu, S. & Werner, F. Distribution and composition of biogenic structures on the Iceland-Faeroe Ridge; relation to different environments. *Palaios* **9**, 92–101 (1994).
25. Barker, P. F., Camerlenghi, A., Acton, G. D. & al., e. *Proc. ODP*, 178 (1999).
26. Bromley, R. G. & Hanken, N. M. Structure and function of large, lobed *Zoophycos*, Pliocene of Rhodes, Greece. *Palaeogeogr. Palaeoclimatol. Palaeoecol.* **192**, 79–100 (2003).
27. Ekdale, A. A. in *Trace fossils 2* (eds Crimes, T. P. & Harper, J. C.) 163–182 (Seel House Press, 1977).
28. Miller III, W. & Vokes, E. H. Large Phymatoderma in Pliocene slope deposits, northwestern Ecuador: associated ichnofauna, fabrication, and behavioral ecology. *Ichnos* **6**, 23–45 (1998).
29. Kotake, N. Paleoecology of the *Zoophycos* producers. *Lethaia* **22**, 327–341 (1989).
30. Kotake, N. Mode of ingestion and egestion of the *Chondrites* and *Zoophycos* producers. *J. Geol. Soc. Jap.* **96**, 859–868 (1990).
31. Kotake, N. Non-selective surface deposit feeding by the *Zoophycos* producers. *Lethaia* **24**, 379–385 (1991).
32. Kotake, N. Deep-sea echiurans: possible producers of *Zoophycos*. *Lethaia* **25**, 311–316 (1992).
33. Kotake, N. Tiering of trace fossil assemblages in Plio-Pleistocene bathyal deposits of Boso Peninsula, Japan. *Palaios* **8**, 544–553 (1993).
34. Kotake, N. Population paleoecology of the *Zoophycos*-producing animal. *Palaios* **9**, 84–91 (1994).
35. Exon, N. *et al.* Morphology, water characteristics and sedimentation in the silled Sulu Sea, southeast Asia. *Mar. Geol.* **39**, 165–195 (1981).
36. Miller, M. F., Cowan, E. A. & Nielsen, S. H. H. Significance of the trace fossil *Zoophycos* in Pliocene deposits, Antarctic continental margin (ANDRILL 1B drill core). *Antart. Sci.* **21**, 609–618 (2009).
37. Hasson, P. F. & Fischer, A. G. Observations on the Neogene of northwestern Ecuador. *Micropaleontol.*, 32–42 (1986).
38. Manley, R. & Lewis, D. Ichnocoenoses of the Mount Messenger Formation, a Miocene submarine fan system, Taranaki Basin, New Zealand. *N.Z. J. Geol. Geophys.* **41**, 15–33 (1998).
39. Blom, W. M. Stratigraphy and sedimentology of Tokomaru Formation (Late Miocene-Early Pliocene), eastern Raukumara Peninsula. *N.Z. J. Geol. Geophys.* **27**, 125–137 (1984).
40. Barron, J., Larsen, B. & al., e. *Proc. ODP* **119, 10.2973/odp.proc.ir.119.1989** (1989).
41. Gani, M. R. & Alam, M. M. Sedimentation and basin-fill history of the Neogene clastic succession exposed in the southeastern fold belt of the Bengal Basin Bangladesh: a high-resolution sequence stratigraphic approach. *Sediment. Geol.* **155**, 227–270 (2003).
42. Encinas, A. *et al.* Rapid and major coastal subsidence during the late Miocene in south-central Chile. *J. South Am. Earth Sci.* **25**, 157–175 (2008).
43. Pudsey, C. J. Asymmetry of *Zoophycus* burrows as a way-up criterion. *Sedimentology* **30**, 301–304 (1983).
44. Uchman, A. Taxonomy and palaeoecology of flysch trace fossils: The Marnoso-arenacea Formation and associated facies (Miocene, Northern Apennines, Italy). *Berigneria* **15**, 3–115 (1995).

45. Le Roux, J. P., Nielsen, S. N. & Henriquez, A. Depositional environment of Stelloglyphus Ilicoensis isp nov.: a new radial trace fossil from the Neogene Ranquil Formation, south-central Chile. *Rev. Geol. Chile* **35**, 307–319 (2008).
46. Tavernier, M. d. A. L'Ichnofacies a *Zoophycos* del <<Flysch DI Faeto>>(Appennino Dauno). Considerazioni paleoambientali. *Mem. Soc. Geol. Ita.* **41**, 605–608 (1988).
47. Uchman, A. & Dem ŕcan, H. A *Zoophycos* Group trace fossil from Miocene flysch in southern Turkey: evidence for a U-shaped causative burrow. *Ichnos* **6**, 251–259 (1999).
48. Demircan, H. & Toker, V. Trace fossils in the western fan of the cingoz formation in the northern andana basin (southern Turkey). *Min. Res. Expl. Bull.* **127**, 15–32 (2003).
49. Donovan, S. K. & Harper, D. A. A new paleobathymetric interpretation of the middle miocene grand bay formation of Carriacou (Grenadines, lesser antilles). *Ichnos* **6**, 283–288 (1999).
50. Pervesler, P., Uchman, A. & Hohenegger, J. New methods for ichnofabric analysis and correlation with orbital cycles exemplified by the Baden-Sooss section (Middle Miocene, Vienna Basin). *Geol. Carp.* **59**, 395–410 (2008).
51. Wagreich, M., Pervesler, P., Khatun, M., Wimmer-Frey, I. & Scholger, R. Probing the underground at the Badenian type locality: geology and sedimentology of the Baden-Sooss section (Middle Miocene, Vienna Basin, Austria). *Geol. Carp.* **59**, 375–394 (2008).
52. Stanley, B. D. Ichnofabric analysis and benthic oxygenation in the Monte dei Corvi cliffs, Ancona, Italy. *Keck Research Symposium in Geology* **9**, 297–300 (1996).
53. Pervesler, P. & Uchman, A. Ichnofossils from the type area of the Grund Formation (Miocene, Lower Badenian) in Northern Lower Austria (Molasse Basin). *Geol. Carp.* **55**, 103–110 (2004).
54. Roetzel, R. & Pervesler, P. Storm-induced event deposits in the type area of the Grund Formation (Middle Miocene, Lower Badenian) in the Molasse Zone of Lower Austria. *Geol. Carp.* **55**, 87–102 (2004).
55. Cabrera, M. I. L., Olivero, E. B., Carmona, N. B. & Ponce, J. J. Cenozoic trace fossils of the *Cruziana*, *Zoophycos* and *Nereites* ichnofacies from the Fuegian Andes, Argentina. *Ameghiniana* **45**, 377–392 (2008).
56. Hayes, D. E., Frakes, L. A. & Shipboard, S. P. *DSDP* **28**, 1017 (1975).
57. Piper, D. J. W. & Brisco, C. B. Deep water continental margin sedimentation, DSDP Leg 28 Antarctica. *DSDP* **28**, 727–755 (1975).
58. Ekdale A A & Bromley R G. Comparative Ichnology of Shelf-Sea and Deep-Sea Chalk. *J. Paleontol.* **58**, 322–332 (1984).
59. Warne, J. E., Kennedy, W. J. & Schneidermann. Biogenic sedimentary structures (trace fossils) in leg 15 cores. *DSDP* **15**, 813–831 (1973).
60. McMillen, J. & Lundberg, N. F. Trace fossil assemblages in Leg 66 sediments. *DSDP* **66**, 653–657 (1982).
61. Barker, P. F., kennett, J. P. & al., e. *Proc. ODP* **113**, 785 (1988).
62. H äntzschel, W. Spreitenbauten (*Zoophycos* Massal.) im Septarienton Nordwest-Deutschlands. *Mitt.Geol.Staatsinst. Hamburg* **29**, 95–100 (1960).
63. Webby, B. D. Trace fossils *Zoophycos* and *Chondrites* from the Tertiary of New Zealand. *N.Z. J. Geol. Geophys.* **12**, 208–214 (1967).
64. Lewis, D. The New Zealand *Zoophycos*. *N.Z. J. Geol. Geophys.* **13**, 295–315 (1970).
65. Chen, W. S. Characteristic trace fossils from shoreface to offshore environments of an oligocene succession, northeastern Taiwan. *T. A. O. S.* **16**, 1097–1120 (2005).
66. Kotlarczyk, J. & Uchman, A. Integrated ichnology and ichthyology of the Oligocene Menilite Formation, Skole and Subsilesian nappes, Polish Carpathians: A proxy to oxygenation history. *Palaeogeogr. Palaeoclimatol. Palaeoecol.* **331**, 104–118 (2012).
67. Malaroda, R. I fossili del Flysch di Annot e la nuova localita fossilifera di Col de Turini (Alpi Marittime). *Boll. Soc. Palaeontol. Ita.* **26**, 11–20 (1987).
68. Bischoff, B. *Zoophycos*, a polychaete annelid, Eocene of Greece. *J. Paleontol.* **42**, 1439–1443 (1968).
69. Harrington, J. Effects of burrowing organisms in deep ocean sediments on the distribution of chemical elements and the preservation and orientation of microfossils. *DSDP* **41**, 933–936 (1977).
70. Mrinjek, E., Pencinger, V., Sremac, J. & Luksic, B. The benkovac stone member of the promina formation: a late eocene succession of storm-dominated shelf deposits. *Geol. Cro.* **8**, 163–184 (2005).
71. Bellotti, N. d. S. P. & Valeri, P. New data on the ecology of *Zoophycos* in pelagic sediments of Garulla, Sibillini Mountains, Marches Apennines. *Bol. Soc. Geol. Ita.* **106**, 813–817 (1987).
72. Barsanti, D. L. Considerazioni sorpa IL Genere *Zoophycos*. *Atti Societa Toscana Scienze Naturrali* **18**, 68–94 (1902).
73. Wetzel, A. Asymmetry of *Zoophycos* burrows as a way-up criterion--a reconsideration. *Sedimentology* **32**, 749–751 (1985).
74. D'Alessandro, A., Ekdal, A. A. & Sonnino, M. Sedimentologic significance of turbidite ichnofacies in the saraceno formation (eocene), southern Italy. *J. Sediment. Petrol.* **56**, 294–306 (1986).
75. L ópez Cabrera, M. I., Olivero, E. B., Carmona, N. B. & Ponce, J. J. Cenozoic trace fossils of the *Cruziana*, *Zoophycos* and *Nereites* ichnofacies from the Fuegian Andes, Argentina. *Ameghiniana* **45**, 377–392 (2008).
76. Buatois, L. A., M ángano, M. G. & Sylvester, Z. A diverse deep-marine Ichnofauna from the Eocene Tarcau sandstone of the Eastern Carpathians, Romania. *Ichnos* **8**, 23–62 (2001).
77. Garcia-Ramos, J. C., Remacha, E., Suarez de Centi, C., Valenzuela, M. & Oms, o. Aportaciones para la interpretacion de *Zoophycos*. *Geogaceta* **15**, 6–9 (1994).
78. Stevens, G. The Amuri fucoid. *N.Z. J. Geol. Geophys.* **11**, 253–261 (1968).
79. Uchman, A. Trace fossils from stress environments in Cretaceous-Paleogene Flysch of the Polish outer Carpathians. *Annales Societatis Geologorum Poloniae* **61**, 207–220 (1991).
80. Ortiz, S. *et al.* Palaeoenvironmental turnover across the Ypresian-Lutetian transition at the Agost section, Southastern Spain: In search of a marker event to define the Stratotype for the base of the Lutetian Stage. *Mar. Micropaleontol.* **69**, 297–313 (2008).
81. Plička, M. Origin of Fossil *Zoophycos*. *Nature* **208**, 579 (1965).
82. Plička, M. *Zoophycos*, and a proposed classification of sabellid worms. *J. Paleontol.* **42**, 836–849 (1968).
83. Plička, M. Methods for the study of *Zoophycos* and similar fossils. *N.Z. J. Geol. Geophys.* **12**, 551–573 (1969).
84. Plička, M. in *Trace fossils* (eds Crimes, T. P. & Harper, J. C.) 361–370 (Seel House Press, 1970).
85. Nielsen, J. K., Gormus, M., Uysal, K. & Kanbur, S. First records of trace fossils from the Lake District, southwestern Turkey. *Bul. Geosci.* **85**, 691–708 (2010).

86. Mohseni, H., Behbahani, R., Khodabakhsh, S. & Atashmard, Z. Depositional environments and trace fossil assemblages in the Pabdeh Formation (Paleogene), Zagros Basin, Iran. *Neues Jahrb. Gel. Palaeontol. Abh.* **262**, 59–77 (2011).
87. Monaco, P., Trecci, T. & Uchman, A. Taphonomy and ichnofabric of the trace fossil *Avetoichnus luisae* Uchman & Rattazzi, 2011 in Paleogene deep-sea fine-grained turbidites: examples from Italy, Poland and Spain. *Boll. Soc. Palaeontol. Ita.* **51**, 23–38 (2012).
88. Olivero, E. B., Lopez, M. I., Malumian, N. & Torres Carbonell, P. J. Eocene graphoglyptids from shallow-marine, high-energy, organic-rich, and bioturbated turbidites, Fuegian Andes, Argentina. *Acta Geol. Pol.* **60**, 77–91 (2010).
89. Tunis, G. & Uchman, A. Trace fossils and facies changes in Cretaceous - Eocene flysch deposits of the Julian Prealps (Italy and Slovenia): Consequences of regional and world-wide changes. *Ichnos* **4**, 169–190 (1996).
90. Giannetti, A. Influence of climate, sea-level changes and tectonics on ichnoassemblages distribution in a carbonate-dominated, deep-marine environment (Upper Paleocene, Zumaya section). *Palaeogeogr. Palaeoclimatol. Palaeoecol.* **285**, 104–118 (2010).
91. Giannetti, A. & McCann, T. The Upper Paleocene of the Zumaya Section (Northern Spain): Review of the Ichnological Content and Preliminary Palaeoecological Interpretation. *Ichnos* **17**, 137–161 (2010).
92. Cummings, J. P. & Hodgson, D. M. Assessing controls on the distribution of ichnotaxa in submarine fan environments, the Basque Basin, Northern Spain. *Sediment. Geol.* **239**, 162–187 (2011).
93. Rodríguez-Tovar, F. J., Uchman, A., Orue-Etxebarria, X. & Apellaniz, E. Palaeoenvironmental changes during the Danian-Selandian boundary interval: The ichnological record at the Sopelana section (Basque Basin, W Pyrenees). *Sediment. Geol.* **284**, 106–116 (2013).
94. Miller III, W. Trace fossil assemblages in Cretaceous-Paleogene pelagic limestones of the Belluno area, northeastern Italy. *Mem. Sci. Geol.* **52**, 175–192 (2000).
95. Miller, W. & D'Alberto, L. Paleoethologic implications of *Zoophycos* from Late Cretaceous and Paleocene limestones of the Venetian Prealps, northeastern Italy. *Palaeogeogr. Palaeoclimatol. Palaeoecol.* **166**, 237–247 (2001).
96. Miller, W. Ⅲ. Paleobiology of complex trace fossils. *Palaeogeogr. Palaeoclimatol. Palaeoecol.* **192**, 3–14 (2003).
97. Carcá-Ramos, J. C., Valenzuela, M., Centi, C. S. D. & Bahamonde, J. R. EI icnogenero *Zoophycos* y su valor sedimentologico y paleoambiental. *Acta Geol. Hisp.* **21–22**, 499–505 (1987).
98. Kedzierski, M., Rodriguez-Tovar, F. J. & Uchman, A. Vertical displacement and taphonomic filtering of nannofossils by bioturbation in the Cretaceous-Palaeogene boundary section at Caravaca, SE Spain. *Lethaia* **44**, 321–328 (2011).
99. Fütterer, D. K. Bioturbation and trace fossils in deep sea sediments of the walvis ridge Southeastern Atlantic, Leg 74. *DSDP* **74**, 543–555 (1984).
100. Pavlovec, R. *Zoophycos* (Annelida, Polychaeta) v podsabotinskih plasteh zahodne Slovenije. *Geologija* **14**, 63–66 (1971).
101. Bradley, J. *Zoophycos* and umbellula (Pennatulacea): Their synthesis and identity. *Palaeogeogr. Palaeoclimatol. Palaeoecol.* **13**, 103–128 (1973).
102. Crimes, T. P. From limestones to distal turbidites: a facies and trace fossil analysis in the Zumaya flysch (Paleocene-Eocene), North Spain. *Sedimentology* **20**, 105–131 (1973).
103. Valeri, N. S. P. Impronte di *Zoophycos* nei calcari marnosi paleogenici al valico delle capannelle (L'Aquila). *Boll. Soc. Geol. It.* **94**, 2155–2182 (1975).
104. Rodríguez-Tovar, F. J. & Uchman, A. Ichnological analysis of the Cretaceous-Palaeogene boundary interval at the Caravaca section, SE Spain. *Palaeogeogr. Palaeoclimatol. Palaeoecol.* **242**, 313–325 (2006).
105. Rodríguez-Tovar, F. J. & Uchman, A. Bioturbational disturbance of the Cretaceous-Palaeogene (K-Pg) boundary layer: Implications for the interpretation of the K-Pg boundary impact event. *Geobios* **41**, 661–667 (2008).
106. Uchman, A., Bubík, M. & Mikuláš, R. The ichnological record across the Cretaceous/Tertiary boundary in turbiditic sediments at Uzgrun (Moravia, Czech Republic). *Geol. Carp.* **56**, 57–65 (2005).
107. Massalongo, A. *Zoophycos, novum genus Plantarum fossilium*. 45–52 (Antonellianis, Veronae, 1855).
108. Ekdale, A. & Lewis, D. The New Zealand *Zoophycos* revisited: morphology, ethology, and paleoecology. *Ichnos* **1**, 183–194 (1991).
109. Lewis, D. W. & Ekdale, A. A. Composite ichnofabric of a mid-Tertiary unconformity on a pelagic limestone. *Palaios* **7**, 222–235 (1992).
110. Rodríguez-Tovar, F. J., Uchman, A., Molina, E. & Monechi, S. Bioturbational redistribution of Danian calcareous nannofossils in the uppermost Maastrichtian across the K-Pg boundary at Bidart, SW France. *Geobios* **43**, 569–579 (2010).
111. Rodríguez-Tovar, F. J., Uchman, A., Orue-Etxebarria, X., Apellaniz, E. & Baceta, J. I. Ichnological analysis of the Bidart and Sopelana Cretaceous/Paleogene (K/Pg) boundary sections (Basque Basin, W Pyrenees): Refining eco-sedimentary environment. *Sediment. Geol.* **234**, 42–55 (2011).
112. Kennedy, W. & Summesberger, H. Lower Maastrichtian ammonites from Neuberg, Steiermark, Austria. *Beiträge zur Paläontologie von Österreich* **12**, 181–242 (1986).
113. Martino, R. L. & Curran, H. A. Sedimentology, ichnology, and paleoenvironments of the Upper Cretaceous Wenonah and Mt. Laurel formations, New Jersey. *J. Sediment. Res.* **60**, 125–144 (1990).
114. Bromley, R. G. & Ekdale, A. A. Trace fossil preservation in flint in the European chalk. *J. Paleontol.* **58**, 298–311 (1984).
115. Ekdale, A. A. & Bromley, R. G. Analysis of composite ichnofabrics: An example in uppermost Cretaceous chalk of Denmark. *Palaios* **6**, 232–249 (1991).
116. Zhou, Z. C., Willems, H. & Zhang, B. G. Marine Cretaceous-Paleogene biofacies and ichnofacies in southern Tibet, China, and their sedimentary significance. *Mar. Micropaleontol.* **32**, 3–29 (1997).
117. Ekdale, A. A. & Stinnesbeck, W. Trace fossils in Cretaceous-Tertiary (KT) boundary beds in northeastern Mexico; implications for sedimentation during the KT boundary event. *Palaios* **13**, 593–602 (1998).
118. Alvarez, W., Asaro, F. & Montanari, A. Iridium profile for 10 million years across the Cretaceous-Tertiary boundary at Gubbio (Italy). *Science* **250**, 1700–1702 (1990).
119. Rodríguez-Tovar, F. J. & Uchman, A. Ichnotaxonomic analysis of the Cretaceous/Palaeogene boundary interval in the Agost section, south-east Spain. *Cret. Res.* **25**, 635–647 (2004).
120. Rodríguez-Tovar, F. J. & Uchman, A. Trace fossils after the K-T boundary event from the Agost section, SE Spain. *Geol. Mag.* **141**, 429–440 (2004).
121. Hollister, C. D., Craddock, C. & al., e. *DSDP* **35**, Washington, DC: government Printing offfice, 930pp. (1976).
122. Saha, O., Shukla, U. K. & Rani, R. Trace Fossils from the Late Cretaceous Lameta Formation, Jabalpur Area, Madhya Pradesh: Paleoenvironmental Implications. *J. Geol. Soc. Ind.* **76**, 607–620 (2010).
123. Kern, J. P. & Warme, J. E. Trace Fossils and Bathymetry of the Upper Cretaceous Point Loma Formation, San Diego, California. *Geol. Soc. Am. Bull.* **85**, 893–900 (1974).
124. Lawrence, M. J. Sedimentology and petrography of early diagenetic chert and dolomite in the Late Cretaceous - early Tertiary Amuri Limestone Group, eastern Marlborough, New Zealand. *N.Z. J. Geol. Geophys.* **36**, 9–25 (1993).

125. Uchman, A. Deep-sea trace fossils from the mixed carbonate-siliciclastic flysch of the Monte Antola Formation (Late Campanian-Maastrichtian), North Apennines, Italy. *Cret. Res.* **28**, 980–1004 (2007).
126. Rajchel, J. & Uchman, A. Ichnology of Upper Cretaceous deep-sea thick-bedded flysch sandstones: Lower Istebna Beds, Silesian Unit (Outer Carpathians, southern Poland). *Geol. Carp.* **63**, 107–120 (2012).
127. Mortimore, R. & Pomerol, B. Stratigraphy and eustatic implications of trace fossil events in the Upper Cretaceous chalk of northern Europe. *Palaios* **6**, 216–231 (1991).
128. Uchman, A. Ichnology of the Rhenodanubian Flysch (Lower Cretaceous-Eocene) in Austria and Germany. *Berigneria* **25**, 67–173 (1999).
129. Locklair, R. E. & Savrda, C. E. Ichnofossil tiering analysis of a rhythmically bedded chalk-marl sequence in the Upper Cretaceous of Alabama. *Lethaia* **31**, 311–321 (1998).
130. Locklair, R. E. & Savrda, C. E. Ichnology of rhythmically bedded Demopolis Chalk (Upper Cretaceous, Alabama); implications for paleoenvironment, depositional cycle origins, and tracemaker behavior. *Palaios* **13**, 423–436 (1998).
131. Shultz, M. R. & Hubbard, S. M. Sedimentology, stratigraphic architecture, and ichnology of gravity-flow deposits partially ponded in a growth-fault-controlled slope minibasin, tres pasos formation (Cretaceous), southern Chile. *J. Sediment. Res.* **75**, 440–453 (2005).
132. Mikulas, R., Skupien, P., Bubik, M. & Vasicek, Z. Ichnology of the Cretaceous Oceanic Red Beds (Outer Western Carpathians, Czech Republic). *Geol. Carp.* **60**, 233–250 (2009).
133. Olivero, E. B. & Malumián, N. Mesozoic-Cenozoic stratigraphy of the Fuegian Andes, Argentina. *Geo. Acta* **6**, 5–18 (2008).
134. Mortimore, R. & Pomerol, B. Correlation of the upper cretaceous white chalk (Turonian to Campanian) in the Anglo-Paris Basin. *Proc. Geol. Ass.* **98**, 97–143 (1987).
135. Frey, R. W. & Howard, J. D. in *Trace fossils* (eds Crimes, T. P. & Harper, J. C.) 141–166 (Seel House Press, 1970).
136. Shimeld, J. W. & MacRae, R. A. Sequence stratigraphic interpretation of regional Upper Cretaceous limestone units, offshore eastern Canada. *Atl.Geol.* **36**, 72 (2000).
137. Savrda, C. E. & Bottjer, D. J. Trace-fossil model for reconstructing oxygenation histories of ancient marine bottom waters: application to Upper Cretaceous Niobrara Formation, Colorado. *Palaeogeogr. Palaeoclimatol. Palaeoecol.* **74**, 49–74 (1989).
138. Vossler, S. M. & George Pemberton, S. Ichnology and paleoecology of offshore siliciclastic deposits in the Cardium Formation (Turonian, Alberta, Canada). *Palaeogeogr. Palaeoclimatol. Palaeoecol.* **74**, 217–239 (1989).
139. Rodríguez-Tovar, F. J., Uchman, A. & Martin-Algarra, A. Oceanic Anoxic Event at the Cenomanian-Turonian boundary interval (OAE-2): ichnological approach from the Betic Cordillera, southern Spain. *Lethaia* **42**, 407–417 (2009).
140. Rodríguez-Tovar, F. J. & Uchman, A. Ichnological data as a useful tool for deep-sea environmental characterization: a brief overview and an application to recognition of small-scale oxygenation changes during the Cenomanian-Turonian anoxic event. *Geo-Marine. lett.* **31**, 525–536 (2011).
141. Rodríguez-Tovar, F. J., Uchman, A., Martin-Algarra, A. & O'Dogherty, L. Nutrient spatial variation during intrabasinal upwelling at the Cenomanian-Turonian oceanic anoxic event in the westernmost Tethys: An ichnological and facies approach. *Sediment. Geol.* **215**, 83–93 (2009).
142. Walker, R. G. An incised valley in the Cardium Formation at Ricinus, Alberta: Reinterpretation as an estuary fill. *Sediment. Fac. Analys.*, 47–74 (1995).
143. Vossler, S. M. & Pemberton, S. G. Skolithos in the Upper Cretaceous Cardium Formation: an ichnofossil example of opportunistic ecology. *Lethaia* **21**, 351–362 (1988).
144. Pemberton, S. & Frey, R. Ichnology of storm-influenced shallow marine sequence: Cardium Formation (Upper Cretaceous) at Seebe, Alberta. *Can. Soc. Petrol. Geol. Mem.* **9**, 281–304 (1984).
145. Uchman, A., Rodríguez-Tovar, F. J., Machaniec, E. & Kedzierski, M. Ichnological characteristics of Late Cretaceous hemipelagic and pelagic sediments in a submarine high around the OAE-2 event: A case from the Rybie section, Polish Carpathians. *Palaeogeogr. Palaeoclimatol. Palaeoecol.* **370**, 222–231 (2013).
146. Uchman, A., Rodríguez-Tovar, F. J. & Oszczypko, N. Exceptionally favourable life conditions for macrobenthos during the Late Cenomanian OAE-2 event: Ichnological record from the Bonarelli Level in the Grajcarek Unit, Polish Carpathians. *Cret. Res.* **46**, 1–10 (2013).
147. Monaco, P., Rodríguez-Tovar, F. J. & Uchman, A. Ichnological analysis of Lateral environmental Heterogeneity within the Bonarelli level (uppermost Cenomanian) in the classical localities near Gubbio, central Apennines, Italy. *Palaios* **27**, 48–54 (2012).
148. Kennedy, W. Trace fossils in the Chalk environment. *Trace fossils. Geol. J., Spec*, 263–282 (1970).
149. Savrda, C. E. in *Approaches in High-Resolution Stratigraphic Paleontology* (ed Harries, P. J.) 129–149 (Kluwer Academic Publishers, 2004).
150. Buatois, L. A. & Lopez Angriman, A. O. The ichnology of a submarine braided channel complex: the Whisky Bay Formation, Cretaceous of James Ross Island, Antarctica. *Palaeogeogr. Palaeoclimatol. Palaeoecol.* **94**, 119–140 (1992).
151. Olivero, E. B., Medina, F. A. & López C, M. I. The stratigraphy of cretaceous mudstones in the eastern Fuegian Andes: new data from body and trace fossils orientales: Nuevos datos de cuerpos y trazas fósiles. *Rev. Aso. Geol. Arg.* **64**, 60–69 (2009).
152. Erba, E. & Premoli Silva, I. Orbitally driven cycles in trace - fossil distribution from the Piobbico core (Late Albian, Central Italy). *Orbital forcing and Cyclic Sequences*, 211–225 (1994).
153. Maceachern, J. A. & Burton, J. A. Firmground *Zoophycos* in the Lower Cretaceous Viking Formation, Alberta: A Distal Expression of the *Glossifungites* Ichnofacies. *Palaios* **15**, 387–398 (2000).
154. Butterworth, P., Crame, J., Howlett, P. & Macdonald, D. Lithostratigraphy of Upper Jurassic-Lower Cretaceous strata of eastern Alexander Island, Antarctica. *Cret. Res.* **9**, 249–264 (1988).
155. Kansha, Y. Ichnofossil *Zoophycos* from the Arita Formation at Yuasa-cho, Wakayama Prefecture, Japan. *J. Geol. Soc. Jap.* **80**, 381–382 (1974).
156. Ineson, J. R. Trace Fossils from a submarine fan-slope apron complex in the Cretaceous of James Ross island, Antarctica. *Br.Antact.Surv.Bull* **74**, 1–16 (1987).
157. Olivero, D. Zoophycos distribution and sequence stratigraphy. Examples from the Jurassic and Cretaceous deposits of southeastern France. *Palaeogeogr. Palaeoclimatol. Palaeoecol.* **123**, 273–287 (1996).
158. Olivero, D. Early Jurassic to Late Cretaceous evolution of *Zoophycos* in the French Subalpine Basin (southeastern France). *Palaeogeogr. Palaeoclimatol. Palaeoecol.* **192**, 59–78 (2003).
159. Olivero, D. & Gaillard, C. in *Trace Fossils: Concepts, Problems, Prospects.* (ed Miller III, W.) 466–477 (Elservier, 2007).
160. Savary, B., Olivero, D. & Gaillard, C. Calciturbidite dynamics and endobenthic colonisation: example from a late Barremian (Early Cretaceous) succession in southeastern France. *Palaeogeogr. Palaeoclimatol. Palaeoecol.* **211**, 221–239 (2004).
161. Gaillard, C. Bioturbation des sediments pelagiques du cretace inferieur dans le Bassin vocontien (Chaines subalpines meridionales, France). *Geobios* **17**, 205–214 (1984).

162. Gaillard, C. & Olivero, D. The Ichnofossil Halimedides in Cretaceous pelagic deposits from the Alps: Environmental and ethological significance. *Palaios* **24**, 257–270 (2009).
163. Lukeneder, A. Taphonomy and stratigraphy of Early Cretaceous ammonoid mass occurrences (Late Valanginian; Northern Calcareous Alps; Upper Austria). *Aust. J. Earth Sci.* **98**, 34–51 (2005).
164. Lukeneder, A. & Rehákov á, D. Lower Cretaceous section of the Ternberg Nappe (Northern Calcareous Alps, Upper Austria): facies changes, biostratigraphy and paleoecology. *Geol. Carp.* **55**, 227–237 (2004).
165. Barbu, V. First notice of the *Zoophycos* in the Upper Valanginian deposits from the Bucegi Mountains (South Carpathians). *Acta Palaeontol. Rom.* **5**, 1–9 (2005).
166. Barbu, V. & Melinte-Dobrinescu, M. C. Latest Jurassic to earliest Cretaceous paleoenvironmental changes in the Southern Carpathians, Romania: regional record of the late Valanginian nutrification event. *Cret. Res.* **29**, 790–802 (2008).
167. Michalík, J. & Šimo, V. A new spreite trace fossil from Lower Cretaceous limestone (Western Carpathians, Slovakia). *Earth and Environmental Science Transactions of the Royal Society of Edinburgh* **100**, 417–427 (2009).
168. Richiano, S., Poire, D. G. & Varela, A. N. Ichnology of the Rio Mayer Formation, Lower Cretaceous, Southwestern Gondwana, Agentina. *Ameghiniana* **50**, 273–286 (2013).
169. Eder, V. G., Zanin, Y. N. & Zamirailova, A. G. Trace fossils of the Upper Jurassic Bazhenov and Georgiev Formations of the West Siberian Plate. *Russ. J. Geol. Geophys.* **44**, 517–524 (2003).
170. Lin, W. Q., Yu, G. M., Wang, C. S. & Lan, B. L. Jurassic Trace fossils and ichnofacies from the Mount Qomolangma Region. *J. Min. Petrol.* **3**, 20–31(in Chinese with English abstract) (1982).
171. Goldring, R., Taylor, A. M. & Hughes, G. W. The application of ichnofabrics towards bridging the dichotomy between siliciclastic and carbonate shelf facies: examples from the Upper Jurassic Fulmar Formation (UK) and the Jubaila Formation (Saudi Arabia). *Proc. Geol. Ass.* **116**, 235–249 (2005).
172. Patel, S. J., Desai, B. G. & Shukla, R. Paleoecological significance of the trace fossils of Dhosa Oolite Member (Jumara Formation), Jhura dome, Mainland Kachchh, Western India. *J. Geol. Soc. Ind.* **74**, 601–614 (2009).
173. Bellotti, N. d. S. P. & Valeri, P. L'Influenza Dell'Ambiente sedimentario sull'assetto elicoidale delle strutture a zoophycos. *Boll. Soc. Geol. It.* **97**, 675–685 (1978).
174. Smith, A. & Juntao, X. Palaeontology of the 1985 Tibet geotraverse, Lhasa to Golmud. *Philos. Trans. R. Soc. London Ser. A.* **327**, 53–105 (1988).
175. Gaillard, C. & Olivero, D. A new paleoecologic interpretation of *Zoophycos* Massalongo, 1855. *C.R.Acad.Sci.Pairs* **316**, 823–830 (1993).
176. Olivero, D. & Atrops, F. The Bathonian-Callovian *Zoophycos* series of the Castellane Arc (South-eastern France) in the platform-basin transition zone: stratigraphy and palaeotectonics. *C.R.Acad.Sci.Paris* **323**, 81–88 (1996).
177. Olivero, D. & Gaillard, C. Paleoecology of Jurassic *Zoophycos* from south-eastern France. *Ichnos* **4**, 249–260 (1996).
178. Fernández-López, S. R. Ammonoid taphonomy, palaeoenvironments and sequence stratigraphy at the Bajocian/Bathonian boundary on the Bas Auran area (Subalpine Basin, south-eastern France). *Lethaia* **40**, 377–391 (2007).
179. Tiraboschi, D. & Erba, E. Calcareous nannofossil biostratigraphy (Upper Bajocian-Lower Bathonian) of the Ravin du Bes section (Bas Auran, Subalpine Basin, SE France): Evolutionary trends of Watznaueria barnesiae and new findings of "Rucinolithus" morphotypes. *Geobios* **43**, 59–76 (2010).
180. Tyszka, J. Paleoenvironmental implications from ichnological and microfaunal analyses of Bajocian spotty carbonates, Pieniny Klippen Belt, Polish Carpathians. *Palaios* **9**, 175–187 (1994).
181. Carvalho, C. N. D. & Rodriguez, N. P. C. Los *Zoophycos* del Bajociense-Bathoniense de la Praia da Mareta (Algarve, Portugal): arquitectura y finalidades en régimen de dominancia ecológica. *Rev. Esp. Paleontol.* **18**, 229–241 (2003).
182. Fürsich, F. T. Environmental distribution of trace fossils in the Jurassic of Kachchh (Western India). *Facies* **39**, 243–272 (1998).
183. Saporta, G. Nouveaux documents relativs aux organismes problematiques des anciennes mers. *Bull. Soc. Geol. Fra.* **15**, 286–302 (1887).
184. De Baets, K., Cecca, F., Guiomar, M. & Verniers, J. Ammonites from the latest Aalenian–earliest Bathonian of La Baume (Castellane area, SE France): palaeontology and biostratigraphy. *Swi. J. Geosci.* **101**, 563–578 (2008).
185. Soussi, M. & Ben Ismaïl, M. Platform collapse and pelagic seamount facies: Jurassic development of central Tunisia. *Sediment. Geol.* **133**, 93–113 (2000).
186. El Arabi, H., Ouahhabi, B. & Charriere, A. Les séries du Toarcien-Aalénien du SW du Moyen-Atlas (Maroc); précisions stratigraphiques et signification paléogéographique. *Bul. Soc. Gel. Fra.* **172**, 723–736 (2001).
187. Fischer-Ooster, C. V. *Die fossilen fucoiden der Schweizer-Alpen, nebst Eroterungen uber deren geologisches Alter*. 41–44+Pl.1a (Huber, 1858).
188. Duarte, L. V., Krautter, M. & Soares, A. F. Bioconstructions à spongiaires siliceux dans le Lias terminal du Bassin lusitanien (Portugal); stratigraphie, sédimentologie et signification paléogéographique. *Bul. Soc. Gel. Fra.* **172**, 637–646 (2001).
189. Monaco, P. Relationships between trace-fossil communities and substrate characteristics in some Jurassic pelagic deposits in the Umbria-Marche basin, Central Italy. *Geobios* **28**, 299–311 (1995).
190. Wieczorek, J. Trace fossils from Fleckenmergel facies (Jurassic) of the Tatra Mts. *Geobios* **28**, 425–431 (1995).
191. Avanzini, M. & Leonardi, G. Dinosauri nel Trentino: il giacimento dei Lavini di Marco. *UCT(Uomo-Città-Territorio)* **214**, 35–41 (1993).
192. Dalla Vecchia, F. M. in *Aspects of Sauropod Paleobiology* (eds Lockley, M. G., Dos Santos, V. F., Meyer, C. A., & Hunt, A. P.) 65–73 (Museu Nacional de História Natural, 1994).
193. Leonardi, G. & Mietto, P. in *Dinosa. Ita.* (eds Leonardi, G. & Mietto, P.) 169–245 (Accademia Editoriale, 2000).
194. Baucon, A. & Avanzini, M. *Zoophycos*-like structures associated with dinosaur tracks in a tidal-flat environment: Lower Jurassic (Southern Alps, Italy). *Studi. Trent. Sci. Nat. Acta Geol.* **83**, 123–131 (2008).
195. Cave, M. P. Occurrence of *Zoophycos* in Oretian rocks of the Murihiku Supergroup, South Otago, New Zealand (Note). *N.Z. J. Geol. Geophys.* **25**, 367–369 (1982).
196. Wetzel, A., Blechschmidt, I., Uchman, A. & Matter, A. Highly diverse ichnofauna in late Triassic deep-sea fan deposits of Oman. *Palaios* **22**, 567–576 (2007).
197. Fordyce, R. *Zoophycos* from the Torlesse Supergroup, North Canterbury, New Zealand (Note). *N.Z. J. Geol. Geophys.* **19**, 289–291 (1976).
198. Zhao, X. *et al.* Trace fossils of the upper triassic bayanharshan group in the Qumarleb-Zhidoi area, southern Qinghai. *Acta Palaeontol. Sin.* **46**, 122–134 (2007).
199. Zhu, B., Zhang, Q. H. & C., T. R. Middle-Late Triassic trace fossils and trace facies from Namda, Zamtang, Sichuan. *Acta Geol. Sic.* **20**, 3–29(in Chinese with English abstract) (2000).
200. Yang, F. Q., ., Wang, Y. P. & Zhu, S. H. The Late Triassic Zhuwo Formation in Maladun, Songpan, Sichuan: Trace fossils and their depositional environments. *Litho. Paleogeogr.* **16**, 34–41(In Chinese with English abstract) (1996).
201. Stockar, R., Adatte, T., Baumgartner, P. O. & Föllmi, K. B. Palaeoenvironmental significance of organic facies and stable isotope signatures: the Ladinian San Giorgio Dolomite and Meride Limestone of Monte San Giorgio (Switzerland, WHL UNESCO).

Sedimentology **60**, 239–269 (2013).
202. Knaust, D. The oldest Mesozoic nearshore *Zoophycos*: evidence from the German Triassic. *Lethaia* **37**, 297–306 (2004).
203. Zhang, G. C. & Li, J. L. Triassic flysch trace fossils and their geological significance from East Karakorum Mountains, China. *Sci. Geol. Sin.* **33**, 257–266(in Chinese with English abstract) (1998).
204. Knaust, D. *Balanoglossites* Magdefrau, 1932 from the Middle Triassic of Germany: part of a complex trace fossil probably produced by burrowing and boring polychaetes. *Palaeontol. Zeit.* **82**, 347–372 (2008).
205. Seilacher, A. Lebensspuren und Salinitatsfazies. *Fortschr. Geol. Rheinld. u. Westf.* **10**, 81–94 (1963).
206. Ma, Z., Yan, J., Xie, X., Ruan, X. & Li, B. Depositional and Ecological Features of Permian Oxygen Deficient Deposits at Shangsi Section, Northeast Sichuan, China. *J. China Univ. Geosci.* **19**, 488–495 (2008).
207. Bhargava, O. N., Srivastava, R. N. & Gadhoke, S. K. *Zoophycos* from the Permian Gungri member (Kuling Formation), Spiti Valley, Himachal Himalaya. *J. Geol. Soc. Ind.* **26**, 137–140 (1985).
208. Knaust, D. The end‐Permian mass extinction and its aftermath on an equatorial carbonate platform: insights from ichnology. *Terra Nova* **22**, 195–202 (2010).
209. Knaust, D. Ichnology as a tool in carbonate reservoir characterization: A case study from the Permian-Triassic Khuff Formation in the Middle East. *Geoarabia* **14**, 17–38 (2009).
210. Wignall, P. B., Morante, R. & Newton, R. The Permo-Triassic transition in Spitsbergen; δ^{13}Corg chemostratigraphy, Fe and S geochemistry, facies, fauna and trace fossils. *Geol. Mag.* **135**, 47–62 (1998).
211. Twitchett, R. J. Palaeoenvironments and faunal recovery after the end-Permian mass extinction. *Palaeogeogr. Palaeoclimatol. Palaeoecol.* **154**, 27–37 (1999).
212. Crowder, R. K. Permian and Triassic Sedimentation in the Northeastern Brooks Range, Alaska: Deposition of the Sadlerochit Group *AAPG Bulletin* **74**, 1351–1370 (1990).
213. Gates, L. M., James, N. P. & Beauchamp, B. A glass ramp: shallow-water permian spiculitic chert sedimentation, sverdrup basin, arctic Canada. *Sediment. Geol.* **168**, 125–147 (2004).
214. Beus, S. S. Fossil associations in the High Tor Limestone (Lower Carboniferous) of South Wales. *J. Paleontol.*, 651–667 (1984).
215. Nakrem, H. A., Szaniawski, H. & MQRK, A. Permian—Triassic scolecodonts and conodonts from the Svalis Dome, central Barents Sea. *Acta Palaeontol. Pol.* **46**, 69–86 (2001).
216. Gong, Y. M., Shi, G. R., Weldon, E. A., Du, Y. S. & Xu, R. Pyrite framboids interpreted as microbial colonies within the Permian *Zoophycos* spreiten from southeastern Australia. *Geol. Mag.* **145**, 95–103 (2008).
217. Shi, G. R., Weldon, E. A. & Pierson, R. R. Permian stratigraphy, sedimentology and paleontology of the southern Sydney Basin, south-east Australia- A field excursion guide (2010 version) prepared for the 6 th International Brachiopod Congress, 1–5 February 2010, Melbourne, Australia. Association of Australasian Palaeontologists Field Guide Series NO.1. *Geol. Soc. Aust.*, 45–48 (2010).
218. Knaust, D. Complex behavioural pattern as an aid to identify the producer of *Zoophycos* from the Middle Permian of Oman. *Lethaia* **42**, 146–154 (2009).
219. Gong, Y. M., Shi, G. R., Zhang, L. J. & Weldon, E. A. *Zoophycos* composite ichnofabrics and tiers from the Permian neritic facies in South China and south-eastern Australia. *Lethaia* **43**, 182–196 (2010).
220. Gong, Y. M. *et al.* Microbial and molecular fossils from the Permian *Zoophycos* in South China. *Sci. Chi. Earth Sci.* **50**, 1121–1127 (2007).
221. Zhang, L., Shi, G. R. & Gong, Y. M. An ethological interpretation of *Zoophycos* based on Permian records from South China and southeastern Australia. *Palaios* **30**, 408–425 (2015).
222. Li, J. Y., Jin, H. J. & Li, Y. C. Trace fossils and their environmental significance of Permian in Laibin and Heshan regions of Guangxi, South China. *Sci. Geol. Sin.* **2**, 143–150 (in Chinese with English abstract) (1986).
223. Reid, C. M., James, N. P., Beauchamp, B. & Kyser, T. K. Faunal turnover and changing oceanography: Late Palaeozoic warm-to-cool water carbonates, Sverdrup Basin, Canadian Arctic Archipelago. *Palaeogeogr. Palaeoclimatol. Palaeoecol.* **249**, 128–159 (2007).
224. Liu, Z. H. Synecology of Lower Permian Qixia Formation in Hunan and Guangxi. *J. Xiangtan Min. Inst.* **7**, 21–30(in Chinese with English abstract) (1992).
225. McGugan, A. Problematical "*Zoophycos*" from the Permian of Western Canada. *Ann. Mag. Nat. Hist.* **6**, 107–112 (1963).
226. McGugan, A. *Zoophycos* from the Permian of Western Canada. *GSA Spe. Paper*, 283–284 (1964).
227. Vaziri, S. H., Yao, A. & Kuwahara, K. Lithofacies and microbiofacies (fora m in ¡fers and radiolarians) of the Permian Sequence in the Shalamzar area, Central Alborz, North Iran. *J. Geosci. Osaka City Univ.* **48**, 39–69 (2005).
228. Sarkar, S., Ghosh, S. K. & Chakraborty, C. Ichnology of a Late Palaeozoic ice-marginal shallow marine succession: Talchir Formation, Satpura Gondwana basin, central India. *Palaeogeogr. Palaeoclimatol. Palaeoecol.* **283**, 28–45 (2009).
229. Hu, B., Liu, S. X., Song, H. B., Wang, Y. Y. & Zhang, L. in *Program and Abstracts with Field Trip Guidebook-The 10th International Ichnofabric workshop, Jiaozuo, China* (eds Gong, Y. M. *et al.*) 84–96 (Henan Polytechnic University 2009).
230. Hu, B., Song, H., Liu, S. & Zhang, L. Sedimentary facies, ichnofossils and storm deposits in the Lower Permian Taiyuan Formation, Jiaozuo city, Henan Province, central China. *Acta Geol. Pol.* **60**, 45–52 (2010).
231. Bhattacharya, B. & Bhattacharya, H. Implications of trace fossil assemblages from Late Paleozoic Glaciomarine Talchir Formation, Raniganj Basin, India. *Gond. Res.* **12**, 509–524 (2007).
232. Chamberlain, C. K. & Clark, D. L. Trace fossils and conodonts as evidence for deep-water deposits in the Oquirrh Basin of central Utah. *J. Paleontol.* **47**, 663–682 (1973).
233. Algeo, T. J., Schwark, L. & Hower, J. C. High-resolution geochemistry and sequence stratigraphy of the Hushpuckney Shale (Swope Formation, eastern Kansas): implications for climato-environmental dynamics of the Late Pennsylvanian Midcontinent Seaway. *Chem. Geol.* **206**, 259–288 (2004).
234. Baucon, A. & Neto de Carvalho, C. From the river to the sea; Pramollo, a new ichnolagerstaette from the Carnic Alps (Carboniferous, Italy-Austria). *Studi. Trent. Sci. Nat. Acta Gel.* **83**, 87–114 (2008).
235. Kabanov, P. The upper Moscovian and basal Kasimovian (Pennsylvanian) of central European Russia: Facies, subaerial exposures and depositional model. *Facies* **49**, 243–270 (2003).
236. Mcllroy, D. & Lang, H. F. Discovery and paleoenvironmental implications of a *Zoophycos*-group trace fossil (? Echinospira) from the Middle Pennsylvanian Sydney Mines Formation of Nova Scotia. *Atl.Geol.* **42**, 31–35 (2006).
237. Tong, Z. S. Trace fossils and their sedimentary environments from Yangbukou Formation of Upper Carboniferous in Jingyuan, Gansu. *Journal of Lanzhou University (Natural Sciences), Geology special issue* **24**, 36–43(in Chinese with English abstract) (1988).
238. Jin, H. J. & Li, Y. C. Carboniferous biogenic sedimentary structures on the northwestern margin of Jungar Basin. *Chin. Sci. Bull.* **44**, 368–372 (1999).

239. Kues, B. S. The trace fossil *Zoophycos* in the Sandia Formation of north-central New Mexico. *New Mex. Geol.* **27**, 70–71 (2005).
240. Martino, R. L. & Sanderson, D. D. Fourier and autocorrelation analysis of estuarine tidal rhythmites, lower Breathitt Formation (Pennsylvanian), eastern Kentucky, USA. *J. Sediment. Res.* **63**, 105–119 (1993).
241. Jin, H. J. & Li, Y. C. Late Paleozoic deep slope ichnofacies and their environmental significance from Jungar Basin. *Sci. Sin. Ser. B* **4**, 409–415 (1991).
242. Miller, M. F. Morphology and paleoenvironmental distribution of Paleozoic *Spirophyton* and *Zoophycos*: implications for the *Zoophycos* ichnofacies. *Palaios* **6**, 410–425 (1991).
243. Liu, Y. & Gastaldo, R. A. Characteristics of a Pennsylvanian ravinement surface. *Sediment. Geol.* **77**, 197–213 (1992).
244. Shao, L. Y. & Liu, Q. F. Trace fossils and their environmental distributions for the Lower Carboniferous of Central Hunan. *J. Stratigra.* **18**, 173–180(in Chinese with English abstract) (1995).
245. Zhang, X., D. Lower Carboniferous trace fossils and their environmental significance from Shuizutang, Lian County, Guangdong Province. *Bull. Nanjing Inst. Gel. M. R., Chinese Acad. Geol. Sci.* **10**, 103–116(in Chinese with English abstract) (1989).
246. Zhang, X. P. & Qing, S. K. Upper Devonian trace fossils from Xinhua country, Hunan and their sedimentary environment. *Oil & Gas geology* **9**, 252–260 (in Chinese with English abstract) (1988).
247. Seilacher, A. Upper Paleozoic trace fossils from the Gilf Kebir-Abu Ras area in southwestern Egypt. *J. Afri.Earth Sci.* **1**, 21–34 (1983).
248. Yang, S. P. The discovery of *Zoophycos* in China and its environmental significance. *Oil & Gas geology* **5**, 228–235 (in Chinese with English abstract) (1984).
249. Yang, S. P. The upper Devonian and Lower Carboniferous trace fossils from Hua county near Guangzhou, China. *J. Ear. Sci.* **32**, 573–580 (in Chinese with English abstract) (1989).
250. Zhang, X. P. & Qing, S. K. Trace fossils and sedimentary environments of Lower Carboniferous at Xinhua, Hunan: Acta Palaeontologica Sinica. *Acta Palaeontol. Sin.* **31**, 605–617 (in Chinese with English abstract) (1992).
251. Wang, S. Y. The discovery of *Zoophycos* in Lower Carboniferous series in Guizhou. *Geology of Guizhou* **6**, 117–122 (in Chinese with English abstract) (1989).
252. Lin, G. The Lower Carboniferous Visan *Zoophycos* from Dayu, Guilin and their palaeoenvironmental significance. *J. Guilin College Geol.* **7**, 199–205 (In Chinese) (1987).
253. Mikuláš, R., Lehotský, T. & Bábek, O. Trace fossils of the Moravice Formation from the southern Nizky Jesenik Mts. (Lower Carboniferous, Culm facies; Moravia, Cezch Republic). *Bul. Geosci.* **79**, 81–98 (2004).
254. Muszer, J. & Haydukiewicz, J. First Paleozoic *Zoophycos* trace fossils from the Sudetes (the Bardo Unit). *Geo. Quart.* **54**, 381–384 (2010).
255. Gaillard, C., Hennebert, M. & Olivero, D. Lower Carboniferous *Zoophycos* from the Tournai area (Belgium): Environmental and ethologic significance. *Geobios* **32**, 513–524 (1999).
256. Fraaye, R. H. B. & Werver, O. P. Trace fossils and their environmental significance in Dinantian carbonates of Belgium. *Palaeont. Z.* **64**, 367–377 (1990).
257. Rodriguez, J. & Gutschick, R. Late Devonian-early Mississippian ichnofossils from western Montana and northern Utah. *Trace fossils. Geol. J. spec*, 407–438 (1970).
258. Wu, X.-t. Storm-generated depositional types and associated trace fossils in Lower Carboniferous shallow-marine carbonates of Three Cliffs Bay and Ogmore-by-Sea, South Wales. *Palaeogeogr. Palaeoclimatol. Palaeoecol.* **39**, 187–202 (1982).
259. Turner, B. R. & Benton, M. J. Paleozoic trace fossils from the Kufra Basin, Libya. *J. Paleontol.* **57**, 447–460 (1983).
260. He, J. S. The trace fossils occurring in the Devonian strata of the Northern Belt in the Eastern Shanyang County of Shanxi and their environmental significance. *Geology of Shanxi* **7**, 34–41(in Chinese with English abstract) (1989).
261. Miller, M. F. Styles of behavioral complexity recorded by selected trace fossils. *Palaeogeogr. Palaeoclimatol. Palaeoecol.* **192**, 33–43 (2003).
262. Zhang, L. J. & Gong, Y. M. New discovery of Late Devonian plant and trace fossils in Hougaoping, Guangyuan, Sichuan. *J. Stratigra.* **33**, 138–146 (In Chinese with English abstract) (2009).
263. Cluff, R. M. Paleoenvironment of the New Albany Shale Group (Devonian-Mississippian) of Illinois. *J. Sediment. Petrol.* **50**, 767–780 (1980).
264. Vanuxem, L. *Geology of New York Part III Survery of the Third Geological District*. 127–178 (1842).
265. Wang, Y. The approach to palaeoecology of ichnofossils *Zoophycos* from middle Devonian in Dushan county, Guizhou province. *Acta Palaeontol. Sin.* **43**, 591–596 (In Chinese with English abstract) (2004).
266. Miller, M. F. Paleoenvironmental distribution of trace fossils in the Catskill deltaic complex, New York State. *Palaeogeogr. Palaeoclimatol. Palaeoecol.* **28**, 117–141 (1979).
267. Plumstead, E. P. A general review of the Devonian fossil plants found in the cape system of south africa. *Palaeontol. Afr.* **10**, 1–83 (1967).
268. Ellenor, D. W. The occurrence of the trace fossil *Zoophycos* in the middle Devonian of northeastern new South Wales, Australia. *Palaeogeogr. Palaeoclimatol. Palaeoecol.* **7**, 69–78 (1970).
269. Sequeira Fernandes, A. C., Medina da Fonseca, V. M. & Martins de Oliveira Ponciano, L. C. Ichnofossils from the Parnaiba Basin: the contributions of Wilhelm Kegel. *Rev. Bra. Paleontol.* **15**, 153–163 (2012).
270. Gong, Y. M. Trace fossils of Early-Middle Devonian clastic Formations in Southwestern Hunan and their relation to sedimentary environments. *Lithofacies Palaeogeography Collection: Beijing, Geological publishing houses* **4**, 98–116 (in Chinese with English abstract) (1987).
271. Zhang, X. P. Trace fossils from Tiaomajian Formation of Middle Devonian in Xinhua, Xiantan and other areas. *Hum. Geol.* **10**, 97–104 (in Chinese with English abstract) (1991).
272. Zhang, X. P. Trace fossils and their sedimentary environments in the middle Devonian Tiaomajian Formation in Xinhua and Xiangtan, Hunan. *Lithofacies Palaeogeography Collection: Beijing, Geological publishing houses* **12**, 34–42 (in Chinese with English abstract) (1992).
273. Chlupáč, I. Structure and environment of the ichnofossil *Zoophycos* in the Lower Devonian of Bohemia, Czechoslovakia. *Casopis pro Mineralogii a Geologii* **35**, 373–387 (1990).
274. Marintsch, E. J. & Finks, R. M. *Zoophycos* size may indicate environmental gradients. *Lethaia* **11**, 273–279 (1978).
275. Chamberlain, C. K. Trace-Fossil Biofacies in the Lower and MIddle Paleozoic of Central Nevada. *Brigham Young University Geology Studies* **26**, 9–19 (1979).
276. Campanha, V. A. The significance of the ichnofossil *Zoophycos* in sedimentation of the ponta grossa formation, Parana Basin. *An Acad. brasil.Cienc.* **57**, 116 (1985).
277. Simões, M. G., de Mello, L. H. C., Rodrigues, S. C., de Moraes Leme, J. & Marques, A. C. Conulariid taphonomy as a tool in paleoenviron mental analysis. *Rev. Bra. Geoc.* **30**, 757–762 (2000).
278. Ruppel, S. C. & Barnaby, R. J. Contrasting Styles of Reservoir Development in Proximal and Distal Chert Facies: Devonian Thirtyone Formation, Texas. *AAPG Bulletin* **85**, 7–33 (2001).

279. Gaillard, C. & Racheboeuf, P. R. Trace fossils from nearshore to offshore environments: Lower Devonian of Bolivia. *J. Paleontol.* **80**, 1205–1226 (2006).
280. Yang, S. P. Early Devonian trace fossils from liujing, Hengxian, Guangxi and their paleoecological signifincance. *Geol. Bul. China* **5**, 11–20 (in Chinese with English abstract) (1983).
281. Palma, F. G. A. R. M. Presencia de *Zoophycos* sp en el silurico del ambiente sudandino de salta. *Acta Geol. Lilo.* **12**, 77–80 (1978).
282. Rubinstein, C. V. & Toro, B. A. Aeronian (Llandovery, Lower Silurian) palynomorphs and graptolites from the Lipeón Formation, Eastern Cordillera, north-west Argentina. *Geobios* **39**, 103–111 (2006).
283. Astini, R. A. & Marengo, L. F. Paleoambientes y estratigrafía secuencial de depósitos marinos marginales del Ordovícico de la sierra de Zapla (Sierras Subandinas, noroeste argentino) y su relación con la Cuenca Andina Central. *Rev. Geol. Chile* **33**, 247–276 (2006).
284. Miller, M. F. Probable inorganic *Zoophycos*-like structure from a Lower Paleozoic Quartzarenite. *J. Paleontol.* **49**, 1127–1129 (1975).
285. Kakuwa, Y. & Webb, J. Trace fossils of a Middle to Upper Ordovician pelagic Deep-ocean bedded chert in Southeastern Australia *Soc. Sediment. Geol.* **88**, 267–276 (2007).
286. Pickerill, R. K., Fillion, D. & Harland, T. Middle Ordovician trace fossils in carbonates of the Trenton Group between Montreal and Quebec City, St. Lawrence lowland, eastern Canada. *J. Paleontol.* **58**, 416–439 (1984).
287. Fei, A.-W. Trace fossil assemblages and palaeoenvironment of Middle Ordovician Gongwusu Formation, Zhouzishan, Inner Mongolia. *Geoscience* **14**, 366–372 (2000).
288. Doucek, J. & Mikuláš, R. Cambrian trace fossil *Zoophycos* from the Czech Republic. *Geol. Carp.* **65**, 403–409 (2014).
289. Yang, S. P. & Wang, X. C. Middle Cambrian Hsuchuangian trace fossils from southern North China platform and their sedimentological significance. *Acta Palaeontol. Sin.* **30**, 74–89 (in Chinese with English abstract) (1991).
290. Goldring, R. & Jensen, S. Trace fossils and biofabrics at the Precambrian–Cambrian boundary interval in western Mongolia. *Geol. Mag.* **133**, 403–415 (1996).
291. Sappenfield, A., Droser, M., Kennedy, M. & Mckenzie, R. The oldest *Zoophycos* and implications for Early Cambrian deposit feeding. *Geol. Mag.* **149**, 1118–1123 (2012).
